# Design of Uniform Hollow Carbon Nanoarchitectures: Different Capacitive Deionization between the Hollow Shell Thickness and Cavity Size

**DOI:** 10.1002/advs.202206960

**Published:** 2023-01-19

**Authors:** Yijian Tang, Jiani Ding, Wenxuan Zhou, Shuai Cao, Feiyu Yang, Yangyang Sun, Songtao Zhang, Huaiguo Xue, Huan Pang

**Affiliations:** ^1^ School of Chemistry and Chemical Engineering Yangzhou University Yangzhou Jiangsu 225002 P. R. China

**Keywords:** capacitive deionization, cavity size, hollow carbon, shell thickness, template method

## Abstract

Carbon‐based materials with high capacitance ability and fast electrosorption rate are ideal electrode materials in capacitive deionization (CDI). However, traditional carbon materials have structural limitations in electrochemical and desalination performance due to the low capacitance and poor transmission channel of the prepared electrodes. Therefore, reasonable design of electrode material structure is of great importance for achieving excellent CDI properties. Here, uniform hollow carbon materials with different morphologies (hollow carbon nanospheres, hollow carbon nanorods, hollow carbon nano‐pseudoboxes, hollow carbon nano‐ellipsoids, hollow carbon nano‐capsules, and hollow carbon nano‐peanuts) are reasonably designed through multi‐step template method and calcination of polymer precursors. Hollow carbon nanospheres and hollow carbon nano‐pseudoboxes exhibit better capacitance and higher salt adsorption capacity (SAC) due to their stable carbonaceous structure during calcination. Moreover, the effects of the thickness of the shell and the size of the cavity on the CDI performance are also studied. HCNSs‐0.8 with thicker shell (≈20 nm) and larger cavity (≈320 nm) shows the best SAC value of 23.01 mg g^−1^ due to its large specific surface area (1083.20 m^2^ g^−1^) and rich pore size distribution. These uniform hollow carbon nanoarchitectures with functional properties have potential applications in electrochemistry related fields.

## Graphical abstract

Homogeneous hollow carbon materials with different morphologies (HCNSs, HCNRs, HCNBs, HCNEs, HCNCs, HCNPs) are reasonably designed, and their capacitive deionization properties are explored. In addition, the influence of shell thickness and cavity size on CDI performance is explored.

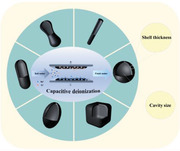

## 1 Introduction

Nanostructured carbon‐based materials have received great attention in various fields, including catalysis, energy storage, adsorption, and biomedicine.^[^ ^1^, ^2^, ^3^ ^]^ This extensive application can be credited to the distinct characteristics of carbon‐based materials including good conductivity, favorable chemical and thermal stability, intrinsic hydrophobicity, and easy surface modification.^[^ ^4^, ^5^, ^6^ ^]^ Among them, hollow carbon materials (HCs) consist of carbon shells and internal cavities, which possess additional features, such as accessible internal cavity, porous carbon shells, high surface area, and adjustable porosity.^[^ ^7^, ^8^, ^9^ ^]^ In particular, the hollow cavity can offer a high specific surface area together with reduced charge and mass diffusion distance and the mesopores on the shell can provide diffusion channels for rapid ion movement.^[^ ^10^, ^11^, ^12^ ^]^ These prominent properties make HCs as a promising candidate for CDI with the superior capacitance ability and electrosorption capacity.^[^ ^13^, ^14^, ^15^ ^]^

Resorcinol‐formaldehyde (RF) resin is commonly used in carbon coating precursor because of its low price, sufficient carbon production and easy activation into pore forming.^[^ ^16^, ^17^, ^18^ ^]^ It is widely used to coat silicon‐based materials,^[^ ^19^, ^20^ ^]^ metal oxides,^[^ ^21^ ^]^ and metal nanoparticles^[^ ^22^ ^]^ and is carbonized into porous carbon shells during the calcination process under inert gas. The thickness of carbon shell can be readily regulated by changing the amount of resorcinol and formaldehyde.^[^ ^23^ ^]^ Template method is a general method to synthesize HCs with uniform pore structure.^[^ ^24^, ^25^, ^26^ ^]^ Up to now, tremendous amounts of HCs has been prepared through template methods and self‐template methods. Different templates have a certain influence on the pore regulation during the formation of RF layer, thus further affecting the pore size distribution of derived carbon layer.^[^ ^27^, ^28^ ^]^ Although self‐template method is considered as a facile strategy for the preparation of HCs, the dynamics process of structure formation is still to be explored.^[^ ^29^ ^]^ In general, the hard template method is easier to be accepted because the morphology or size of the HCs can be well controlled by adjusting the templates.^[^ ^30^, ^31^, ^32^ ^]^ The hollow structures obtained by template method are mostly spherical, and the methods for controlling the synthesis of uniform HCs with other morphologies are quite limited, because it is difficult to form uniform coatings on high curvature surfaces.^[^ ^33^ ^]^ Therefore, it is very meaningful to explore uniform HCs with different morphologies.

Herein, we have synthesized uniform hollow carbon nanospheres (HCNSs), hollow carbon nanorods (HCNRs), hollow carbon nano‐pseudoboxes (HCNBs), hollow carbon nano‐ellipsoids (HCNEs), hollow carbon nano‐capsules (HCNCs), and hollow carbon nano‐peanuts (HCNPs) through multi‐step template methods. First, a series of uniformly dispersed templates were synthesized, including SiO_2_ spheres, MnO_x_ nanowires, and Fe_2_O_3_ particles with different morphologies (pseudocube, ellipsoid, capsule, and peanut). Subsequently, the core–shell structure was synthesized by coating RF resin on the hard template core. Lastly, HCs with different morphologies are obtained through calcination and etching. The structural characterization and electrochemical capabilities of the synthesized HCs were studied comprehensively. Compared with HCs with other morphologies, HCNSs exhibit the best CDI performance (Table S1, Supporting Information), which can be ascribed to the larger specific surface area and better maintenance of carbonaceous during calcination. In addition, the influence of shell thickness and cavity size of HCNSs on CDI was explored. HCNSs‐0.8, which has a thicker shell and a larger cavity, shows a large specific surface area and rich hierarchical pores, resulting in the highest SAC value of 23.01 mg g^−1^. Therefore, the design of appropriate shell thickness and cavity size of uniform HCs with large specific surface area and abundant pore distribution can broaden the road of prepared carbon‐based materials in the application of CDI.

## 2 Results and Discussion

**Figure 1:**
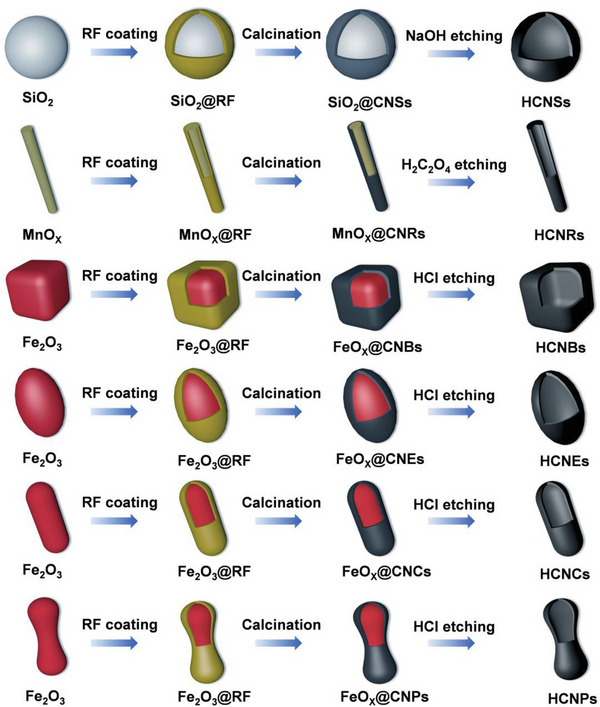
Schematic illustration of the synthesis processes of HCNSs, HCNRs, HCNBs, HCNEs, HCNCs, and HCNPs.

**Figure 2:**
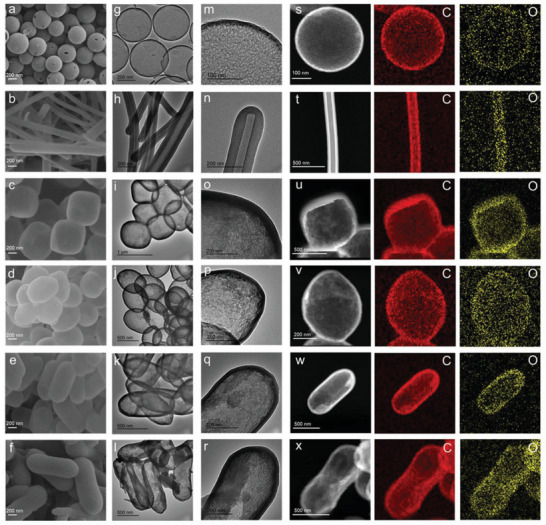
SEM images of a) HCNSs, b) HCNRs, c) HCNBs, d) HCNEs, e) HCNCs, f) HCNPs. TEM images of g,m) HCNSs, h,n) HCNRs, i,o) HCNBs, j,p) HCNEs, k,q) HCNCs, l,r) HCNPs. STEM images and EDX elemental mappings of s) HCNSs, t) HCNRs, u) HCNBs, v) HCNEs, w) HCNCs, x) HCNPs.

The multi‐step process of preparing HCNSs, HCNRs, HCNBs, HCNEs, HCNCs, HCNPs are shown in **Figure** **1**. First, uniform SiO_2_ nanospheres, MnO_x_ nanowires (Figure S1, Supporting Information), Fe_2_O_3_ pseudocubes (Figure S2, Supporting Information), Fe_2_O_3_ ellipsoids (Figure S3, Supporting Information), Fe_2_O_3_ capsules (Figure S4, Supporting Information), and Fe_2_O_3_ peanuts (Figure S5, Supporting Information) were prepared and acted as sacrificing templates. Second, RF resin was carried out on the surface of SiO_2_ nanospheres, MnO_x_ nanowires, Fe_2_O_3_ pseudocubes, Fe_2_O_3_ ellipsoids, Fe_2_O_3_ capsules, and Fe_2_O_3_ peanuts, forming SiO_2_@ RF, MnO_x_@ RF, Fe_2_O_3_@ RF core–shell structures. Afterward, spherical SiO_2_@ RF, rodlike MnO_x_@ RF, pseudocubic Fe_2_O_3_@ RF, ellipsoidal Fe_2_O_3_@ RF, capsule Fe_2_O_3_@ RF, and peanut like Fe_2_O_3_@ RF were calcined at 700 °C in N_2_ atmosphere and converted into SiO_2_@CNSs, MnO_x_@CNRs, FeO_x_@CNBs, FeO_x_@CNEs, FeO_x_@CNCs, and FeO_x_@CNPs, respectively (Figure S6, Supporting Information). It is obvious that the SiO_2_ template has no change after calcination, while the FeO_x_ and MnO_x_ templates are slightly damaged due to the reaction with carbon during calcination. Last, the SiO_2_ template from SiO_2_@CNSs composite was completely removed by NaOH solution to obtain HCNSs (**Figure** **2**a,g,m). The MnO_x_ template from MnO_x_@CNRs composite was removed by H_2_C_2_O_4_ solution to fabricate HCNRs (Figure 2b,h,n). The FeO_x_ templates from FeO_x_@CNBs, FeO_x_@CNEs, FeO_x_@CNCs, and FeO_x_@CNPs composites were removed by HCl solution to get HCNBs (Figure 2c,i,o), HCNEs (Figure 2d,j,p), HCNCs (Figure 2e,k,q), and HCNPs (Figure 2f,l,r), respectively. Both the scanning electron microscopy (SEM) and transmission electron microscopy (TEM) images indicate that the pores in the carbon shell are disordered. Scanning transmission electron microscopy (STEM) images and energy‐dispersive X‐ray (EDX) elemental mappings of HCNSs, HCNRs, HCNBs, HCNEs, HCNCs, and HCNPs display the existence and distribution of C, and O elements (Figure 2s–x). The thickness of carbon shell is obviously determined by the thickness of RF resin layer. With the regulation with the amount of resorcinol (*R*) and formaldehyde (*F*), the thickness of RF resin layer can be easily controlled. As shown in Figure S7 (Supporting Information), HCNSs with shell thickness of ≈10, 15, and 25 nm (named as HCNSs‐0.2, HCNSs‐0.4, HCNSs‐0.6) were prepared by adjusting the amount of R (0.2, 0.4, 0.6 g) and F (0.28, 0.56, 0.84 mL). The size of cavity is similar to those of template core. Considering the stability of silica core during calcination and the easy regulation of ethyl silicate (TEOS) amount, different sizes of SiO_2_@C were prepared to obtain HCNSs (marked as HCNSs‐0.1, HCNSs‐0.4, HCNSs‐0.8) with different sizes of cavities (≈240, 300, and 320 nm) shown in Figure S8 (Supporting Information). With the increasing amount of TEOS, more initial silica seeds contributed to the increasing hydrolytic rate.^[^ ^34^ ^]^ More short chains of TEOS have been produced, in which these micro‐seeds are unstable in the solvents, causing more frequent collisions between the micro‐seeds. This facilitated the formation of larger nanoparticles.^[^ ^35^ ^]^ Meanwhile, with the increase of TEOS content, the surface of HCNSs becomes rougher and rougher. It is speculated that the porosity in the carbon shell is greatly affected. The pore channels of HCNSs‐0.8 in TEM images (Figure S8k,l, Supporting Information) are easier to observe than those of other HCNSs.

**Figure 3:**
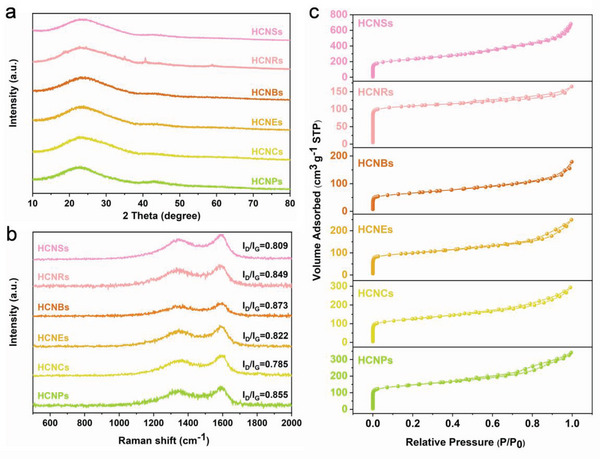
a) XRD patterns, b) Raman plots, and c) N_2_ adsorption–desorption isotherms of HCNSs, HCNRs, HCNBs, HCNEs, HCNCs, and HCNPs.

The crystal structure is analyzed by X‐ray diffraction (XRD) charts in **Figure** **3**a and Figure S9 (Supporting Information). All HCs show two broad peaks at about 22° and 43°, indexed as (002) and (100) planes of the amorphous carbon frameworks.^[^ ^36^ ^]^ The structures of HCNSs, HCNRs, HCNBs, HCNEs, HCNCs, and HCNPs were farther researched by the Raman measurements. As illustrated in Figure 3b, and Figures S10–S11 (Supporting Information), two broad peaks at ≈1360 and 1590 cm^−1^ marked as D band and G band can be observed in HCs. The *I*_D_/*I*_G_ values of HCNSs, HCNRs, HCNBs, HCNEs, HCNCs, and HCNPs are 0.809, 0.849, 0.873, 0.822, 0.785, and 0.855, respectively. Obviously, the selection of the template exerts a certain effect on the *I*_D_/*I*_G_ of hollow carbon materials carbonized by RF resin. In addition, the *I*_D_/*I*_G_ values of HCNSs prepared with SiO_2_ as the template are also different due to different amounts of TEOS and RF resin. The *I*_D_/*I*_G_ values of HCNSs‐0.1, HCNSs‐0.2, HCNSs‐0.4, HCNSs‐0.6, and HCNSs‐0.8 are 0.831, 0.877, 0.809, 0.882, and 0.974, respectively. It strongly demonstrates that the *I*_D_/*I*_G_ values of HCNSs that adjust the carbon shell thickness through the regulation with the amount of RF resin has no obvious change. However, the *I*_D_/*I*_G_ values of HCNSs that regulate the cavity size by simultaneously changing the amount of TEOS and RF resin have obvious differences. The highest *I*_D_/*I*_G_ value of HCNSs‐0.8 suggests that much more defects and disordered degree in the carbon matrix, which promote charge accumulation in the adsorption process.^[^ ^37^ ^]^

The specific surface areas (SSAs) and pore size distribution of the HCs were identified by N_2_ adsorption–desorption isotherms. All the N_2_ adsorption–desorption isotherms (Figure 3c, Figure S12, Table S2, Supporting Information) of HCs show classical shape of type IV, which imply a hierarchical pore structure (Figure S13, Supporting Information) with both micropores and mesopores.^[^ ^38^ ^]^ The SSAs of HCNSs, HCNRs, HCNBs, HCNEs, HCNCs, and HCNPs are 783.84, 342.21, 219.42, 325.78, 412.60, and 476.94 m^2^ g^−1^, respectively. It is visible that the SSA of HCNSs prepared with SiO_2_ as the template are superior to those of the HCs with other morphologies prepared with MnO_X_ and Fe_2_O_3_ as the templates. Moreover, the total pore volumes of HCNSs, HCNRs, HCNBs, HCNEs, HCNCs, and HCNPs are 1.03, 0.25, 0.25, 0.38, 0.45, and 0.52 cm^3^ g^−1^, respectively. These lead to a conclusion that the porosities of HCNSs is higher than those of HCs with other morphologies. As shown in Figure S14 and Table S3 (Supporting Information), the SSAs of HCNSs increase slightly with the increase of the shell thickness, but the total pore volume is basically similar (≈1.0 cm^3^ g^−1^). Meanwhile, with the increase of the cavity size, both the SSAs and the total pore volume of HCNSs increase. HCNSs‐0.8 shows the largest SSA (1083.20 m^2^ g^−1^) and pore volume (2.01 cm^3^ g^−1^), which further validates the above conjecture obtained by SEM and TEM. In addition, more mesopores exist in HCNSs‐0.8 (Figure S14d, Supporting Information), which may be due to a large number of SiO_2_ primary particles participating in auxiliary pore forming. The larger SSA and abundant mesopores provided more active sites and diffusion paths for ion adsorption and transport during the CDI process.^[^ ^39^ ^]^

Figures S15–S20 (Supporting Information) show the X‐ray photoelectron spectroscopy (XPS) analysis of all HCs. As shown in Figures S15 and S18 (Supporting Information), there are two peaks located at ≈285, 534 eV in the full spectrum of all HCs, corresponding to C and O species, respectively. The C1s can clearly obtain four peaks of all HCs: C–C (284.8 ± 0.2 eV); C–O (286.3 ± 0.2 eV); C = O (288.8 ± 0.2 eV), and *π*−*π* conjugate (290.7 ± 0.2 eV). The C–C peak with the highest strength implies that the RF resin precursor is absolutely transformed into HCs.^[^ ^40^ ^]^ From the weak *π*−*π* bond peak, it can be seen that the crystal structure of double‐bond conjugated system is relatively insufficient.^[^ ^41^ ^]^ Therefore, these HCs can be classified as amorphous materials. In the O 1s spectra, all HCs show three O species located at 533.9 ± 0.2, 532.7 ± 0.2, and 531.2 ± 0.2 eV, corresponding to H–O–H, C–O, and C = O groups, respectively. The presence of O contributes to the wettability of HCs and makes the electrolyte easy to penetrate.

**Figure 4:**
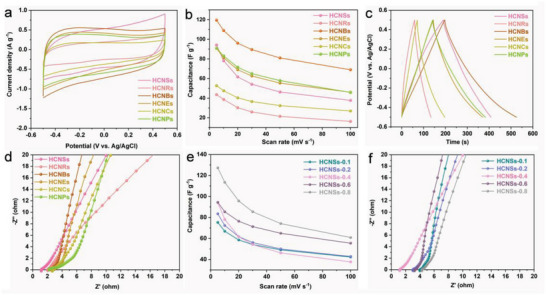
a) CV diagrams at 5 mV s^−1^, b) the corresponding specific capacitances versus scan rates, c) GCD diagrams at 0.5 A g^−1^, and d) EIS of HCNSs, HCNRs, HCNBs, HCNEs, HCNCs, and HCNPs. e) The specific capacitances versus scan rates and f) EIS of HCNSs‐0.1, HCNSs‐0.2, HCNSs‐0.4, HCNSs‐0.6, and HCNSs‐0.8 in 1.0 m NaCl solution.

The electrochemical performance of all HCs electrodes was explored in 1.0 m NaCl solution. **Figure** **4**a exhibits the cyclic voltammetry (CV) curves of HCNSs, HCNRs, HCNBs, HCNEs, HCNCs, and HCNPs electrodes at 5 mV s^−1^. In general, all electrodes show the CV curves with quasi‐rectangular shape, indicative of the capacitive behavior.^[^ ^14^ ^]^ Once the scan rate is raised from 5 to 100 mV s^−1^ (Figures S21 and 22, Supporting Information), the CV curves of all HCs electrodes can still maintain the initial appearance, proving a fine capacitive property as a result of the rapid ions diffusion in the hierarchical hollow porous structures. The specific capacitances of HCNSs, HCNRs, HCNBs, HCNEs, HCNCs, and HCNPs electrodes calculated by Equation (S1) (Supporting Information) are 94.17, 43.61, 119.47, 90.82, 52.68, and 90.78 F g^−1^, respectively. The specific capacitances of HCNSs, HCNRs, HCNBs, HCNEs, HCNCs, and HCNPs electrodes were further shown in Figure 4b at different scan rates. It is apparent that the specific capacitance weakens in the wake of the enhancement of corresponding scanning rate. Furthermore, the galvanostatic charge/discharge (GCD) plots (Figures S23 and S24, Supporting Information) of all HCs electrodes exhibit approximately symmetrical triangle, which are strong evidence of their dominant electric double layer (EDL) behaviors.^[^ ^42^ ^]^ Figure 4c shows the GCD curves of HCNSs, HCNRs, HCNBs, HCNEs, HCNCs, and HCNPs electrodes at 0.5 A g^−1^. Obviously, the discharge time sequence of all HCs electrodes is HCNBs>HCNSs > HCNPs > HCNEs > HCNCs > HCNRs. The discharge time of HCNBs and HCNSs electrodes are longer than that of other HCs electrodes. This trend is consistent with the specific capacitance inferred from CV curves. This may be due to the fact that the carbonaceous structures of HCNBs and HCNSs are not damaged much during the calcination process. While other HCs react with the sacrificial template during the calcination process, resulting in the damage of the carbonaceous structure to a certain extent. The electrochemical resistance of the three‐electrode system was measured by electrochemical impedance spectroscopy (EIS). As exhibited in Figure 4d,f, the lines at the low‐frequency region all show well linearity, representing the dominant behavior of EDL.^[^ ^43^ ^]^ The steepest line of HCNBs electrode indicates the best capacitance performance. The Warburg resistances (*Rw*) can be judged by the slope of the linear curve. The linear curve of HCNBs electrode possesses the larger slope, proving the smaller *Rw* and the rapider charge transport.^[^ ^44^ ^]^ The bulk resistances (*Rs*) can be signified by the *x*‐intercept in the high‐frequency region. The *x*‐intercept of the HCNSs electrode is obviously smaller than that of other HCs electrodes, implying the lower intrinsic resistance of HCNSs (Figure 4d). The specific capacitances of HCNSs‐0.1, HCNSs‐0.2, HCNSs‐0.4, HCNSs‐0.6, and HCNSs‐0.8 electrodes were further shown in Figure 4e at different scan rates. In terms of shell thickness, HCNSs‐0.6 with the thickest shell thickness shows the largest specific capacitance of 94.41 F g^−1^ at 5 mV s^−1^. The specific capacitance of HCNSs‐0.4 is greater than that of HCNSs‐0.2 at low scan rates. Following the improvement of scan rate, the specific capacitance of HCNSs‐0.4 decreases rapidly and is finally lower than that of HCNSs‐0.2. This may be due to the large *Rw* of HCNSs‐0.4 (Figure 4f), and the ions have no enough time to diffuse at high scan rates. Considering the cavity, the specific capacitance at 5 mV s^−1^ of the HCNSs‐0.8 (127.31 F g^−1^) with largest cavity is greater than HCNSs‐0.4 (94.17 F g^−1^) and HCNSs‐0.1 (75.33 F g^−1^) owing to its high SSA and abundant pore size distribution. Similar to the above case of shell thickness, the specific capacitance of HCNSs‐0.4 is greater than HCNSs‐0.1 at low scan rates. Nevertheless, with the increase of scan rates, the specific capacitance of HCNSs‐0.4 is finally less than HCNSs‐0.1. Apparently, HCNSs with thicker shell thickness and larger cavity can produce larger SSA and richer pore size distribution, thus showing better electrochemical performance, which can ensure superior CDI performance.

**Figure 5:**
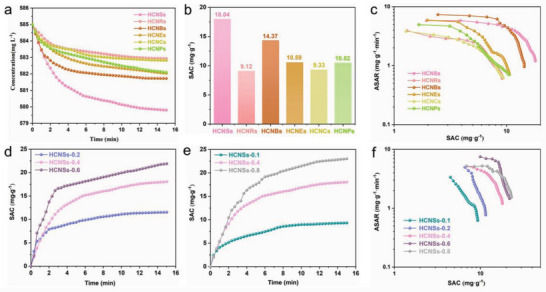
a) CDI profiles, b) desalination capacities, and c) the corresponding CDI Ragone plots of HCNSs, HCNRs, HCNBs, HCNEs, HCNCs, and HCNPs in 10.0 mm NaCl. d) SAC‐time plots of HCNSs‐0.2, HCNSs‐0.4, HCNSs‐0.6. e) SAC‐time plots of HCNSs‐0.1, HCNSs‐0.4, HCNSs‐0.8. f) CDI Ragone plots of HCNSs‐0.1, HCNSs‐0.2, HCNSs‐0.4, HCNSs‐0.6, and HCNSs‐0.8.

The salt adsorption properties of HCNSs, HCNRs, HCNBs, HCNEs, HCNCs, and HCNPs electrodes were investigated in 10.0 mm NaCl at 1.2 V. As shown in **Figure** **5**a, the solution concentration of HCNSs, HCNRs, HCNBs, HCNEs, HCNCs, and HCNPs electrodes decreases significantly in the first 5 min, then slowly decreases, and turns to a relatively stable value within 15 min, revealing that salt ions are adsorbed rapidly and easily. It can be calculated that the SAC value of HCNSs is 18.04 mg g^−1^ (Figure 5b), which is higher than those of HCs with other morphologies (HCNRs: 9.12 mg g^−1^, HCNBs: 14.37 mg g^−1^, HCNEs: 10.59 mg g^−1^, HCNCs: 9.33 mg g^−1^, and HCNPs: 10.52 mg g^−1^). The corresponding Ragone plots of all HCs electrodes are illustrated in Figure 5c. Commonly, the upper and right shifts of the graph can indicate that the electrode has strong electrosorption capacity and fast electrosorption rate. The Ragone plot curve of HCNBs sample is located in the uppest region. The Ragone plot curve of HCNCs sample is located at the far right. Obviously, HCNBs shows the fastest adsorption rate in the initial process of electrosorption. As the adsorption process progresses, the adsorption rate of HCNSs is gradually higher than that of HCNBs. In another word, the HCNSs electrode can remove the most ions in the shorter time. For the purpose of studying the effect of shell thickness and cavity size on HCs in CDI, the electrosorption capacities of HCNSs‐0.1, HCNSs‐0.2, HCNSs‐0.6, and HCNSs‐0.8 were also tested. Figure S25 (Supporting Information) shows the NaCl concentration variations of HCNSs with different shell thickness during electrosorption process. Evidently, the corresponding NaCl concentration of HCNSs‐0.6 with the thickest shell decreases the most. As shown in Figure 5d, the SAC of HCNSs‐0.6 is 21.89 mg g^−1^, which exceedes those of HCNSs‐0.4 (18.04 mg g^−1^), and HCNSs‐0.2 (11.53 mg g^−1^). Meanwhile, Figure S26 (Supporting Information) shows the NaCl concentration variations of HCNSs with different cavity sizes during electrosorption process. The reduction of NaCl concentration of HCNSs‐0.8 electrode is the largest. Thus, HCNSs‐0.8 electrode shows the highest SAC value of 23.01 mg g^−1^ (Figure 5e). Figure 5f shows that HCNSs‐0.8 with thicker shells and larger cavities can remove more ions in a shorter time. As the operating voltage increases from 0.8 to 1.2 V (Figure S27, Supporting Information), the SAC value improves from 8.28 to 23.01 mg g^−1^, and the maximum salt adsorption rate (SAR) value improves from 3.57 to 5.19 mg g^−1^ min^−1^. At an appropriate higher operating voltage, the electrostatic interaction between ions and electrode materials will become more violent, thereby adsorbing greater mass of NaCl. In addition, the cycle stability of the HCNSs‐0.8 electrode in CDI has been explored in 10 mm NaCl solution. Obviously, the desalination capacity of HCNSs‐0.8 electrode exhibits no significant decline after 15 consecutive cycles (Figure S28, Supporting Information). After the electrosorption–desorption cycles, the size, shape, and structural integrity of the carbon skeleton are unchanged for HCNSs‐0.8 sample shown in TEM images and XRD pattern (Figures S29 and 30, Supporting Information), confirming the good cycling stability.

## 3 Conclusion

To conclude, uniform hollow carbon nanoarchitectures (HCNSs, HCNRs, HCNBs, HCNEs, HCNCs, and HCNPs) can be controllably synthesized by multi‐step template methods and calcination of polymer precursors. The selection of templates and the control of etching conditions are important factors for the formation of regular uniform hollow morphology. All HCs are nanoscale amorphous porous carbon materials with well uniform dispersion characteristics. HCNSs and HCNBs exhibit better electrochemical performance and CDI performance compared with other HCs, because they maintain good carbonaceous structure during calcination. HCNSs and HCNBs show better SAC of 18.04, 14.37 mg g^−1^, respectively, at 1.2 V in 10.0 mm NaCl. In order to consider the effect of shell thickness and cavity size on the CDI performance of HCs, HCNSs with different shell thickness (HCNSs‐0.2, HCNSs‐0.4, and HCNSs‐0.6) and different cavity size (HCNSs‐0.1, HCNSs‐0.4, and HCNSs‐0.8) were explored. The final results show that HCNSs‐0.8 with thicker shell and larger cavity displays the highest SAC value of 23.01 mg g^−1^ because of its large specific surface area and abundant hierarchical pores. Hence, designing uniform hollow carbon nanoarchitectures with large specific surface area and abundant hierarchical pores can improve the potential of preparing carbon‐based materials in CDI applications.

## Conflict of Interest

The authors declare no conflict of interest.

## Supporting information

Supporting Information

## Data Availability

The data that support the findings of this study are available in the supplementary material of this article.

## References

[1] M. Kim , R. Xin , J. Earnshaw , J. Tang , J. P. Hill , A. Ashok , A. K. Nanjundan , J. Kim , C. Young , Y. Sugahara , J. Na , Y. Yamauchi , Nat. Protoc. 2022, 17, 2990. 36064756 10.1038/s41596-022-00718-2

[2] D. S. Bin , Z. X. Chi , Y. Li , K. Zhang , X. Yang , Y. G. Sun , J. Y. Piao , A. M. Cao , L. J. Wan , J. Am. Chem. Soc. 2017, 139, 13492. 28858501 10.1021/jacs.7b07027

[3] Q. Wu , X. Yan , Y. Jia , X. Yao , EnergyChem 2021, 3, 100059.

[4] Q. Sun , D. Chen , Q. Huang , S. Huang , J. Qian , Sci. China Mater. 2022.

[5] H. Zhang , O. Noonan , X. Huang , Y. Yang , C. Xu , L. Zhou , C. Yu , ACS Nano 2016, 10, 4579. 27050771 10.1021/acsnano.6b00723

[6] L. Duan , C. Wang , W. Zhang , B. Ma , Y. Deng , W. Li , D. Zhao , Chem. Rev. 2021, 121, 14349. 34609850 10.1021/acs.chemrev.1c00236

[7] Y. Xia , T. Zhao , X. Zhu , Y. Zhao , H. He , C. T. Hung , X. Zhang , Y. Chen , X. Tang , J. Wang , W. Li , D. Zhao , Nat. Commun. 2021, 12, 2973. 34016965 10.1038/s41467-021-23150-8 PMC8137936

[8] W. Wang , B. Xu , X. Pan , J. Zhang , H. Liu , Angew. Chem., Int. Ed. Engl. 2021, 60, 7802. 33404175 10.1002/anie.202014895

[9] W. Xiong , H. Li , H. You , M. Cao , R. Cao , Natl. Sci. Rev. 2020, 7, 609. 34692080 10.1093/nsr/nwz166 PMC8288918

[10] M. Kim , X. Xu , R. Xin , J. Earnshaw , A. Ashok , J. Kim , T. Park , A. K. Nanjundan , W. A. El‐Said , J. W. Yi , J. Na , Y. Yamauchi , ACS Appl. Mater. Interfaces 2021, 13, 52034. 10.1021/acsami.1c09107 34459576

[11] J. Zhang , J. Fang , J. Han , T. Yan , L. Shi , D. Zhang , J. Mater. Chem. A 2018, 6, 15245.

[12] X. Song , D. Fang , S. Huo , X. Song , M. He , W. Zhang , K. Li , Sep. Purif. Technol. 2021, 278, 119550.

[13] X. Xu , S. Zhang , J. Tang , L. Pan , M. Eguchi , J. Na , Y. Yamauchi , EnergyChem 2020, 2, 100043.

[14] F. Yang , S. Cao , Y. Tang , K. Yin , Y. Gao , H. Pang , J. Colloid Interface Sci. 2022, 628, 236. 35940138 10.1016/j.jcis.2022.07.153

[15] X. Xu , J. Tang , Y. V. Kaneti , H. Tan , T. Chen , L. Pan , T. Yang , Y. Bando , Y. Yamauchi , Mater. Horiz. 2020, 7, 1404.

[16] G. H. Kim , W. H. Choi , J. W. Choi , K. H. Kim , D. G. Park , M. G. Park , M. G. Kim , H. Jang , U. H. Kim , J. K. Kang , ACS Nano 2022, 16, 6552. 35377611 10.1021/acsnano.2c00922

[17] J. S. Fang , Y. W. Zhang , Y. M. Zhou , S. Zhao , C. Zhang , C. H. Yang , W. X. Chen , M. Q. Huang , Y. Gao , Carbon 2017, 121, 602.

[18] H. Gao , S. Zuo , S. Wang , F. Xu , M. Yang , X. Hu , Carbon 2022, 194, 220.

[19] D. Liu , X. Huang , D. Qu , D. Zheng , G. Wang , J. Harris , J. Si , T. Ding , J. Chen , D. Qu , Nano Energy 2018, 52, 1.

[20] Y. Wang , Z. Jiao , M. Wu , K. Zheng , H. Zhang , J. Zou , C. Yu , H. Zhang , Nano Res. 2017, 10, 2966.

[21] W. Liu , P. T. Duan , H. W. Xiong , H. L. Su , X. B. Zhang , J. Z. Wang , F. Y. Yang , Z. Q. Zou , J. Mater. Chem. C 2021, 9, 5505.

[22] L. Ding , M. Zhang , Y. Ren , J. Xu , J. Zheng , H. Alsulami , M. A. Kutbi , F.‐Y. Zhang , ACS Appl. Nano Mater. 2020, 3, 4623.

[23] Y. Tang , S. Zheng , S. Cao , F. Yang , X. Guo , S. Zhang , H. Xue , H. Pang , J. Colloid Interface Sci. 2022, 626, 1062. 35839675 10.1016/j.jcis.2022.07.034

[24] J. Liu , T. Yang , D.‐W. Wang , G. Q. Lu , D. Zhao , S. Z. Qiao , Nat. Commun. 2013, 4, 2798.

[25] H. Zhao , F. Zhang , S. Zhang , S. He , F. Shen , X. Han , Y. Yin , C. Gao , Nano Res. 2018, 11, 1822.

[26] Q. Yue , J. Li , Y. Zhang , X. Cheng , X. Chen , P. Pan , J. Su , A. A. Elzatahry , A. Alghamdi , Y. Deng , D. Zhao , J. Am. Chem. Soc. 2017, 139, 15486. 29016118 10.1021/jacs.7b09055

[27] F. Wang , B. Wang , T. Ruan , T. Gao , R. Song , F. Jin , Y. Zhou , D. Wang , H. Liu , S. Dou , ACS Nano 2019, 13, 12219. 31589407 10.1021/acsnano.9b07241

[28] H. X. Wu , H. J. Lv , Y. Zhang , J. Du , A. B. Chen , Inorg. Chem. Front. 2020, 7, 2548.

[29] F. Liu , Y. Cheng , J. Tan , J. Li , H. Cheng , H. Hu , C. Du , S. Zhao , Y. Yan , M. Liu , Front Chem 2021, 9, 668336. 33859976 10.3389/fchem.2021.668336 PMC8042251

[30] Y. Liu , X.‐Y. Yu , Y. Fang , X. Zhu , J. Bao , X. Zhou , X. W. Lou , Joule 2018, 2, 725.

[31] X. Zang , Z. Fu , D. Wang , Z. Yuan , N. Shi , Z. Yang , Y.‐M. Yan , J. Mater. Chem. A 2022, 10, 9988.

[32] M. Ezzati , F. Hekmat , S. Shahrokhian , H. E. Unalan , Desalination 2022, 533, 115766.

[33] Y. Wang , X. Su , S. Lu , J. Mater. Chem. 2012, 22, 1969.

[34] S. Bhattacharya , A. B. Mallick , M. Dutta , S. K. Srivastava , P. Prathap , C. M. S. Rauthan , AIP Conf. Proc. 2020, 2265, 030072.

[35] Y. Yan , J. Fu , L. Xu , T. Wang , X. Lu , Nano Lett 2016, 11, 885.

[36] S. Zhao , T. Yan , H. Wang , G. Chen , L. Huang , J. Zhang , L. Shi , D. Zhang , Appl. Surf. Sci. 2016, 369, 460.

[37] Y. Li , J. Qi , J. Li , J. Shen , Y. Liu , X. Sun , J. Shen , W. Han , L. Wang , ACS Sustainable Chem. Eng. 2017, 5, 6635.

[38] Y. Huang , J. Yang , L. Hu , D. Xia , Q. Zhang , Y. Liao , H. Li , W. Yang , C. He , D. Shu , Environ. Sci.: Nano 2019, 6, 1430.

[39] N. Liu , L. Yu , B. Liu , F. Yu , L. Li , Y. Xiao , J. Yang , J. Ma , Adv. Sci. 2022, 2204041, 10.1002/advs.202204041. PMC9839853 36442852

[40] Y. Li , Y. Liu , J. Shen , J. Qi , J. Li , X. Sun , J. Shen , W. Han , L. Wang , Desalination 2018, 430, 45.

[41] H. Zhang , F. Zhang , Y. Wei , Q. Miao , A. Li , Y. Zhao , Y. Yuan , N. Jin , G. Li , ACS Appl. Mater. Interfaces 2021, 13, 21217. 33909973 10.1021/acsami.1c01137

[42] S. Cao , T. Chen , S. Zheng , Y. Bai , H. Pang , Small Methods 2021, 5, 2101070. 10.1002/smtd.202101070 34928014

[43] H. Zhang , W. Zhang , J. Shen , Y. Li , X. Yan , J. Qi , X. Sun , J. Shen , W. Han , L. Wang , J. Li , Desalination 2020, 473, 114173.

[44] K. Wang , Y. Liu , Z. Ding , Z. Chen , X. Xu , M. Wang , T. Lu , L. Pan , Chem. Eng. J. 2022, 433, 133578.

