# Supplementary material for: Design of Uniform Hollow Carbon Nanoarchitectures: Different Capacitive Deionization between the Hollow Shell Thickness and Cavity Size
Source: Adv Sci (Weinh). 2023 Jan 19;10(9):2206960. doi: 10.1002/advs.202206960 (PMC10037972; doi:10.1002/advs.202206960)
Supplement: Supplementary file 1 — Supporting Information [file ADVS-10-2206960-s001.pdf]

ADVANCED SCIENCE
Open Access

Supporting Information

for *Adv. Sci.*, DOI 10.1002/advs.202206960

# Design of Uniform Hollow Carbon Nanoarchitectures: Different Capacitive Deionization between the Hollow Shell Thickness and Cavity Size

*Yijian Tang, Jiani Ding, Wenxuan Zhou, Shuai Cao, Feiyu Yang, Yangyang Sun, Songtao Zhang, Huaiguo Xue and Huan Pang**

# Supporting Information

**Design of Uniform Hollow Carbon Nanoarchitectures: Different Capacitive Deionization Between the Hollow Shell Thickness and Cavity Size**

*Yijian Tang, Jiani Ding, Wenxuan Zhou, Shuai Cao, Feiyu Yang, Yangyang Sun, Songtao Zhang, Huaiguo Xue, Huan Pang**

Y. Tang, J. Ding, W. Zhou, S. Cao, F. Yang, Y. Sun, S. Zhang, H. Xue, H. Pang
School of Chemistry and Chemical Engineering, Yangzhou University, Jiangsu 225002, China.
E-mail: huanpangchem@hotmail.com; panghuan@yzu.edu.cn

## Experimental Section

***Materials***

All reagents were analytical grade and could be used without further purification. Resorcinol was purchased from Shanghai Aladdin Bio-Chem Technology Co., Ltd. Tetraethyl orthosilicate was purchased from Shanghai Macklin Biochemical Co., Ltd. Formaldehyde, Iron trichloride hexahydrate, Sodium sulfate, Potassium permanganate, $NH_3 \cdot H_2O$, ethanol, NaOH, Poly(1,1-difluoroethylene) (PVDF), N-methyl 2-pyrrolidinone (NMP) were purchased from Sinopharm Chemical Reagent Co., Ltd. Polyvinylpyrrolidone (K23-27) was purchased from Shanghai MERYER Chemical Technology Co., Ltd. Vulcan XC 72 was purchased from Cabot Corporation Agar-agar. All aqueous solutions were freshly prepared with high purity water.

***Synthesis of hollow carbon nanospheres (HCNSs)***

Resorcinol-formaldehyde (RF) oligomers and ethyl silicate (TEOS) were co-condensed on $SiO_2$ core particles to form the $SiO_2$@RF core-shell structured nanospheres. In a typical synthesis of $SiO_2$@RF, 3.46 mL of TEOS was added to the solution containing 70 mL of ethanol, 10 mL of $H_2O$, and 3 mL of $NH_3 \cdot H_2O$ (25

wt %) under vigorous stirring at room temperature. After 15 min, 0.4 g of resorcinol (R) and 0.56 mL of formaldehyde (F) were added to the solution, and the system was kept under vigorous stirring for 24 h at room temperature. The precipitates were separated by centrifugation, washed with deionized water and ethanol, and then dried at 50 $^{o}$C overnight. Second, the precipitates were calcinated under $N_2$ at 700 $^{o}$C for 2 h to obtain the $SiO_2$@CNSs. The obtained $SiO_2$@CNSs were etched in 3M NaOH solution to obtain HCNSs. This sample is also named HCNSs-0.4. Same as the above steps, in order to prepare HCNSs with different shell thicknesses, the amount of R and F is changed to 0.2 g, 0.28 mL (HCNSs-0.2); 0.6 g, 0.84 mL (HCNSs-0.6). Similarly, in order to prepare HCNSs with different cavity sizes, the quantities of TEOS, R and F are changed to 0.865 mL, 0.1 g, 0.14 mL (HCNSs-0.1); 6.92 mL, 0.8 g, 1.12 mL (HCNSs-0.8).

***Synthesis of hollow carbon nanorods (HCNRs)***

$MnO_x$ nanowires were fabricated according to previous report. Briefly, 190 mg of $KMnO_4$ and 100 mg of PVP were dissolved in 80 mL of $H_2O$. After vigorous magnetic stirring at room temperature for 30 min, the resulting solution was transferred into a 100 mL Teflon-lined autoclave, which was sealed and maintained at 160 $^{o}$C for 9 h. After cooling down to room temperature, the resultant $MnO_x$ nanowires were collected by centrifugation, washed with DI water and ethanol several times, and dried at 60 $^{o}$C overnight. 100 mg of $MnO_x$ nanowires were dispersed in the solution containing 32 mL of ethanol, 80 mL of $H_2O$, and 0.4 mL of $NH_3 \cdot H_2O$ by ultrasonic method. Subsequently, 0.4 g of R was added to the solution under vigorous stirring at room temperature. After 30 min, 0.56 mL F was added, and the system was kept under vigorous stirring for 24 h at room temperature. The precipitates were separated by centrifugation, washed with deionized water and ethanol, and then dried at 50 $^{o}$C overnight. Second, the precipitates were calcinated under $N_2$ at 700 $^{o}$C for 2 h to obtain the $MnO_x$@CNRs. The obtained $MnO_x$@CNRs were etched in 0.5 M oxalic acid solution to obtain HCNRs.

***Synthesis of hollow carbon nano-pseudoboxes (HCNBs)***

Pseudocubic $Fe_2O_3$ was fabricated according to previous report. A NaOH solution (90 mL, 6.0 M) was added to 100 mL of well-stirred 2.0 M $FeCl_3 \cdot 6H_2O$ in a 250 mL Pyrex bottle for 30 min. The tightly stoppered bottle containing the Fe $(OH)_3$ gel was placed in a laboratory oven preheated to 100 °C, and the gel was aged for 8 days. After the treatment, red products were collected by filtration and washed three times with deionized water and ethanol before drying at 50 °C overnight. The obtained pseudocubic $Fe_2O_3$ (1.0 g) was dispersed in the solution containing 32 mL of ethanol, 80 mL of $H_2O$, and 0.4 mL of $NH_3 \cdot H_2O$ by ultrasonic method. Subsequently, 0.4 g of R was added to the solution under vigorous stirring at room temperature. After 30 min, 0.56 mL F was added, and the system was kept under vigorous stirring for 24 h at room temperature. The precipitates were separated by centrifugation, washed with deionized water and ethanol, and then dried at 50 $^oC$ overnight. Second, the precipitates were calcinated under $N_2$ at 700 $^oC$ for 2 h to obtain the $FeO_x$@CNBs. The obtained $FeO_x$@CNBs were etched in 2 M HCl solution to obtain HCNBs.

***Synthesis of hollow carbon nano-ellipsoids (HCNEs)***

The process of preparing ellipsoidal $Fe_2O_3$ is similar to that of preparing pseudocubic $Fe_2O_3$. A NaOH solution (90 mL, 6.0 M) was added to 100 mL of well-stirred 2.0 M $FeCl_3 \cdot 6H_2O$ in a 250 mL Pyrex bottle for 5 min, followed by the addition of $Na_2SO_4$ solution (10 mL, 0.20 M), and the agitation was continued for an additional 25 min. The tightly stoppered bottle containing the Fe $(OH)_3$ gel was placed in a laboratory oven preheated to 100 °C, and the gel was aged for 8 days. After the treatment, red products were collected by filtration and washed three times with deionized water and ethanol before drying at 50 °C overnight. Later, the process of preparing HCNEs is the similar to that of preparing HCNBs. The only difference is that the ellipsoidal $Fe_2O_3$ template replaces the pseudocubic $Fe_2O_3$ template.

***Synthesis of hollow carbon nano-capsules (HCNCs)***

The process of preparing rodlike $Fe_2O_3$ is similar to that of preparing ellipsoidal $Fe_2O_3$. The only difference is that the added $Na_2SO_4$ solution concentration is 0.60 M. Later, the process of preparing HCNCs is the similar to that of preparing HCNBs. The

only difference is that the rodlike $Fe_2O_3$ template replaces the pseudocubic $Fe_2O_3$ template.

***Synthesis of hollow carbon nano-peanuts (HCNPs)***

The process of preparing peanut like $Fe_2O_3$ is similar to that of preparing ellipsoidal $Fe_2O_3$. The only difference is that the added $Na_2SO_4$ solution concentration is 1.00 M. Later, the process of preparing HCNPs is the similar to that of preparing HCNBs. The only difference is that the peanut like $Fe_2O_3$ template replaces the pseudocubic $Fe_2O_3$ template.

***Materials characterization***

The morphological features were characterized by field emission scanning electron microscopy (FESEM, Zeiss-Supra55), high resolution transmission electron microscopy (HRTEM, Tecnai G2 F30 S-TWIN), and energy dispersive X-ray spectrometry (EDS) mapping. X-ray diffraction (XRD) patterns were examined on a Bruker D8 Advanced X-ray Diffractometer (Cu-Kα radiation: $\lambda$ = 0.15406 nm). Raman spectroscopy was obtained by using Renishaw InVia Reflex (514 nm laser). The chemical states are measured using an Axis Ultra X-ray photoelectron spectroscope (XPS, Kratos Analytical Ltd., UK) equipped with a standard monochromatic Al-Kα source (hv = 1486.6 eV). Nitrogen sorption isotherms were carried out using a BELSORP-mini (BEL, Japan). The specific surface area (SSA) was analyzed by Multipoint Brunauer-Emmett-Teller (BET) technique.

***Electrochemical performance measurements***

The electrode ink was prepared by mixing 80 wt% active material with 10 wt% Vulcan XC 72 and 10 wt% PVDF in NMP solvent under ultrasonication for 30 min. A certain volume of the ink was dropped onto the graphite paper with a thickness of 0.5 mm (area of $1 \times 1$ $cm^2$) and dried at 60 °C for 12 h. The potential sweep cyclic voltammetry (CV) and electrochemical impedance spectroscopy (EIS) measurements were conducted by using an electrochemical workstation (CHI-760E) with

three-electrode configuration in 1.0 M NaCl electrolyte. The Ag/AgCl electrode and platinum (Pt) wire were used as reference and counter electrodes, respectively. Cyclic voltammetry (CV) and gravimetric charge-discharge (GCD) measurements were carried out in the potential range of -1 to -0.1 V.

The specific capacitance of electrodes was calculated using Eq. (S1),

$$C_m = (\int I dV)/2v\,\Delta V m \qquad \textbf{Eq. (S1)}$$

where $C_m$ (F $g^{-1}$) represents for the specific capacitance of the electrode, $I$ (A) for the current density, $\Delta V$ for the voltage change, $v$ (mV $s^{-1}$) for the scan rate and $m$ (g) for the mass of the working electrode.

***CDI performance measurements***

Membrane assisted CDI (MCDI) unit-cell was constructed with two pairs of identical electrodes, ion exchange membrane for anion and cation, and spacer. Anion- and cation exchange membranes were used to alleviate the co-ion effect. Each individual CDI carbon electrode was composed of active material, Vulcan XC 72, and PVDF in the ratio of 8:1:1, and prepared on the titanium plate of 2×2 $cm^2$ (thickness: 1 mm) as current collector. Before assembling the MCDI unit-cell, the electrodes were immersed in 584 mg/L of saline water for 24 h to completely wet the surface. The CDI tests were conducted using a batch-mode with a continuous recycling system, which includes a CDI cell, a peristaltic pump, a power source, and a fluid reservoir. The ambient temperature and the total volume of the NaCl solution in the desalination experiment were maintained at 298 K and 50 mL, respectively. In the CDI desalination process， the saline water was desalinated through MCDI unit-cell and recycled in a closed circuit. The real-time change of the brackish water concentration was measured by a conductivity probe which was connected to the CDI system. The correlation between conductivity and concentration was achieved based on a calibration table prepared before the test (**Figure S31**). The original concentration of NaCl aqueous solution used in the desalination system is 10 mM (corresponding to 584 mg $L^{-1}$). The applied voltage at both ends of the electrodes is 1.2 V. The salt

adsorption capacity (SAC, mg $g^{-1}$) and average salt adsorption rates (ASAR, mg $g^{-1}$ $min^{-1}$) at t min were calculated as follows:

$$SAC = (C_0 - C_t) \times V/m$$ **Eq. (S2)**

$$\mathrm{ASAR} = SAC/t$$ **Eq. (S3)**

where $C_0$ and $C_t$ are the NaCl concentrations at initial stage and $t$ min, respectively (mg $L^{-1}$), $V$ is the volume of the NaCl solution (L), and $m$ is the total mass of the electrode materials (g).

**Supplementary Figures: Figure S1- S31**

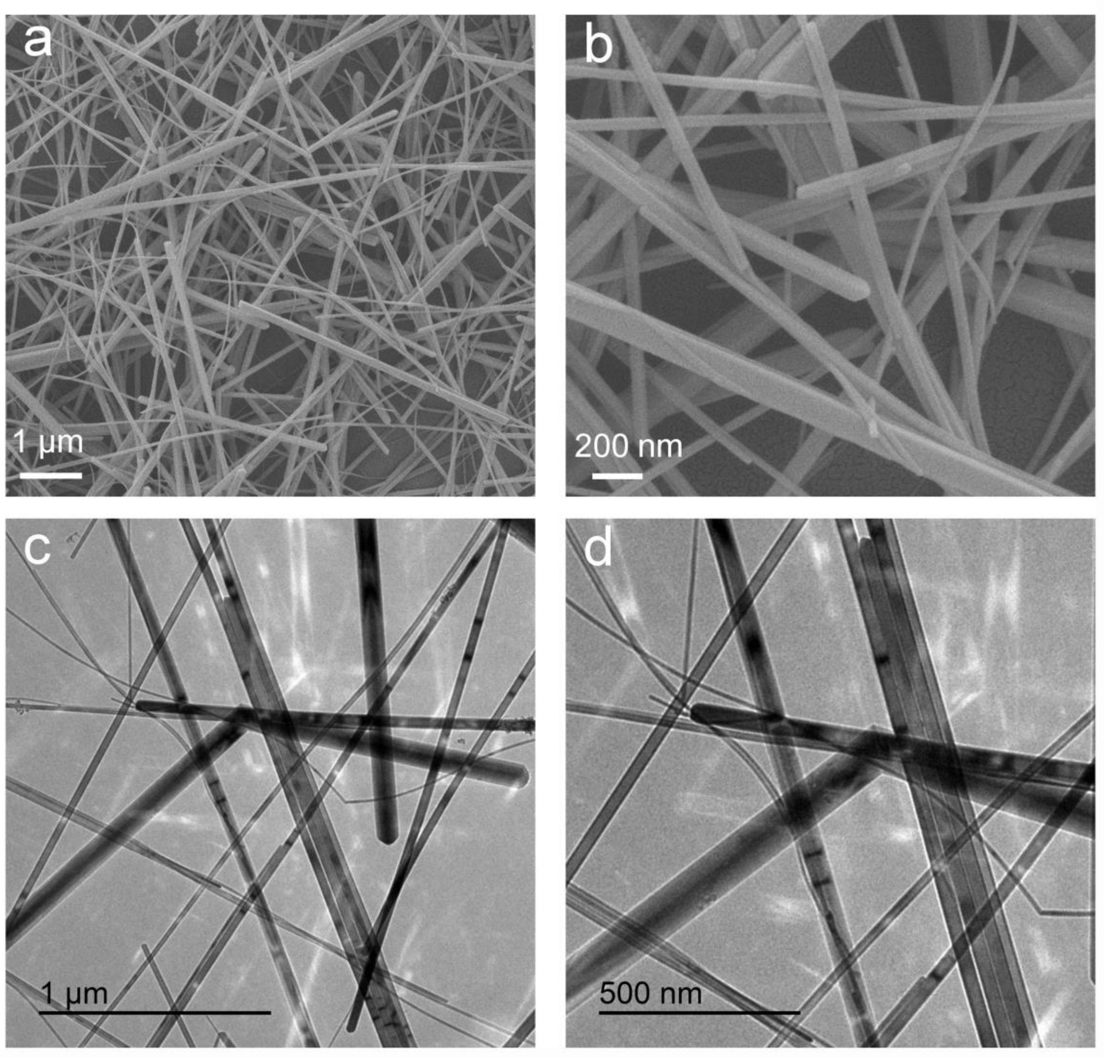


**Figure S1.** (a, b) SEM images of $MnO_x$ nanowires. (c, d) TEM images of $MnO_x$ nanowires.

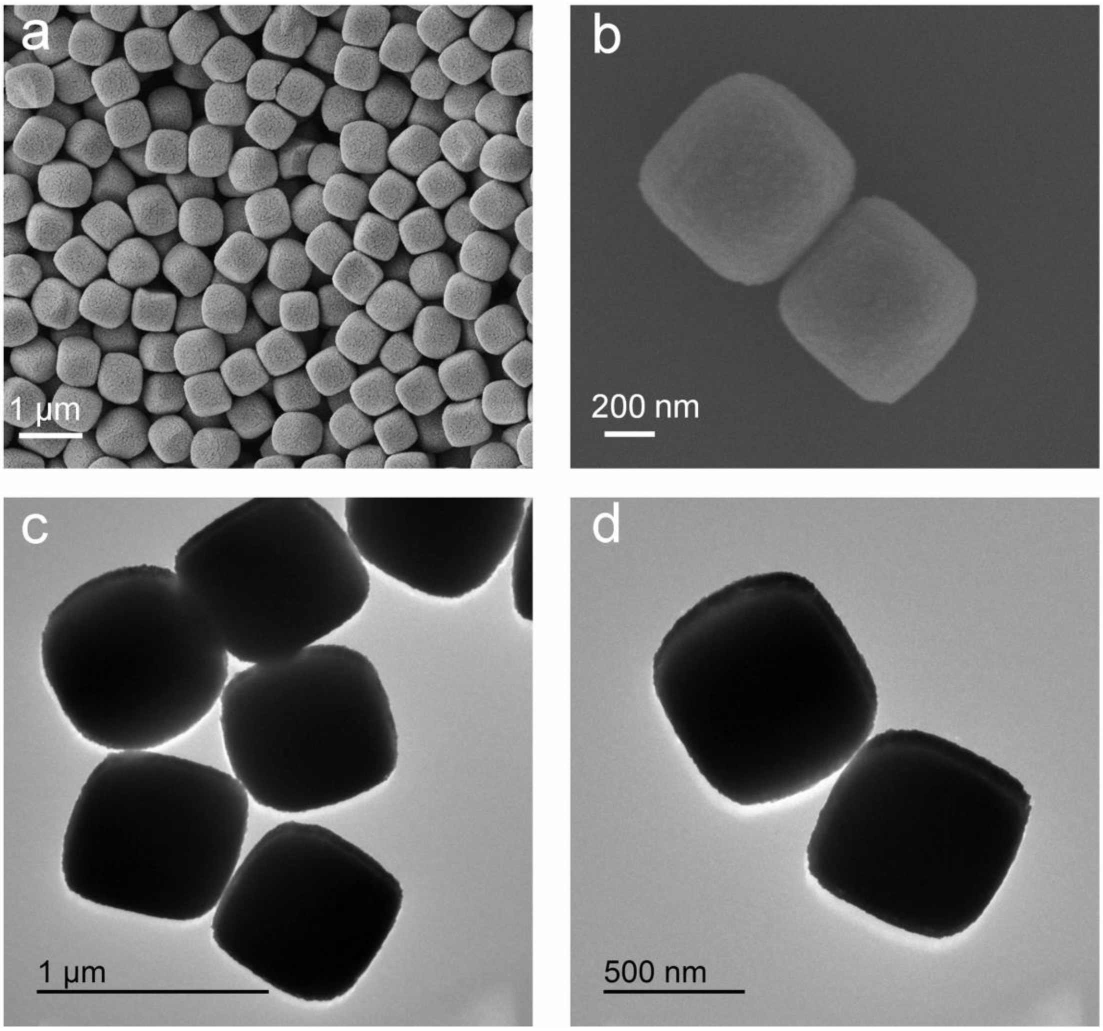


**Figure S2.** (a, b) SEM images of pseudocubic $Fe_2O_3$. (c, d) TEM images of pseudocubic $Fe_2O_3$.

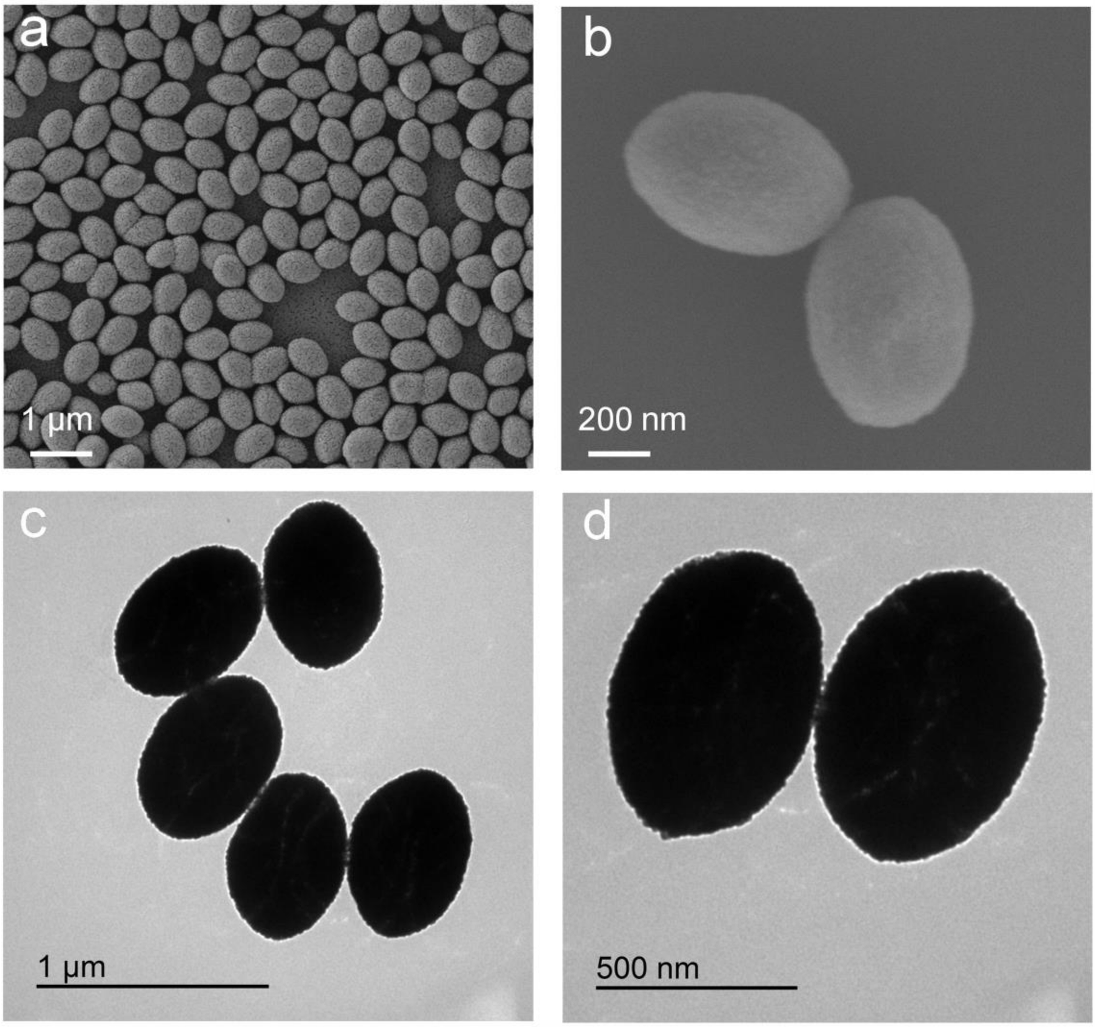


**Figure S3.** (a, b) SEM images of ellipsoidal $Fe_2O_3$. (c, d) TEM images of ellipsoidal $Fe_2O_3$.

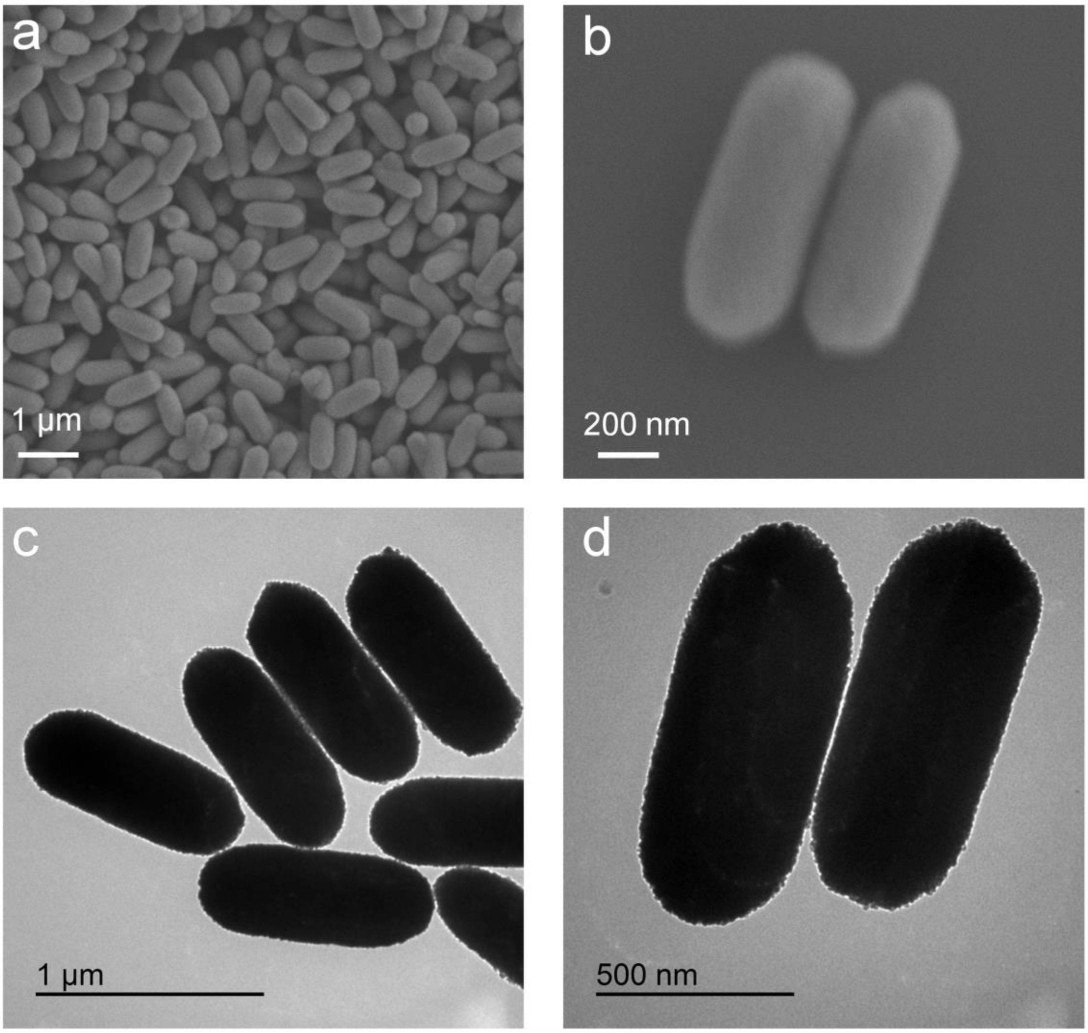


**Figure S4.** (a, b) SEM images of rodlike $Fe_2O_3$. (c, d) TEM images of rodlike $Fe_2O_3$.

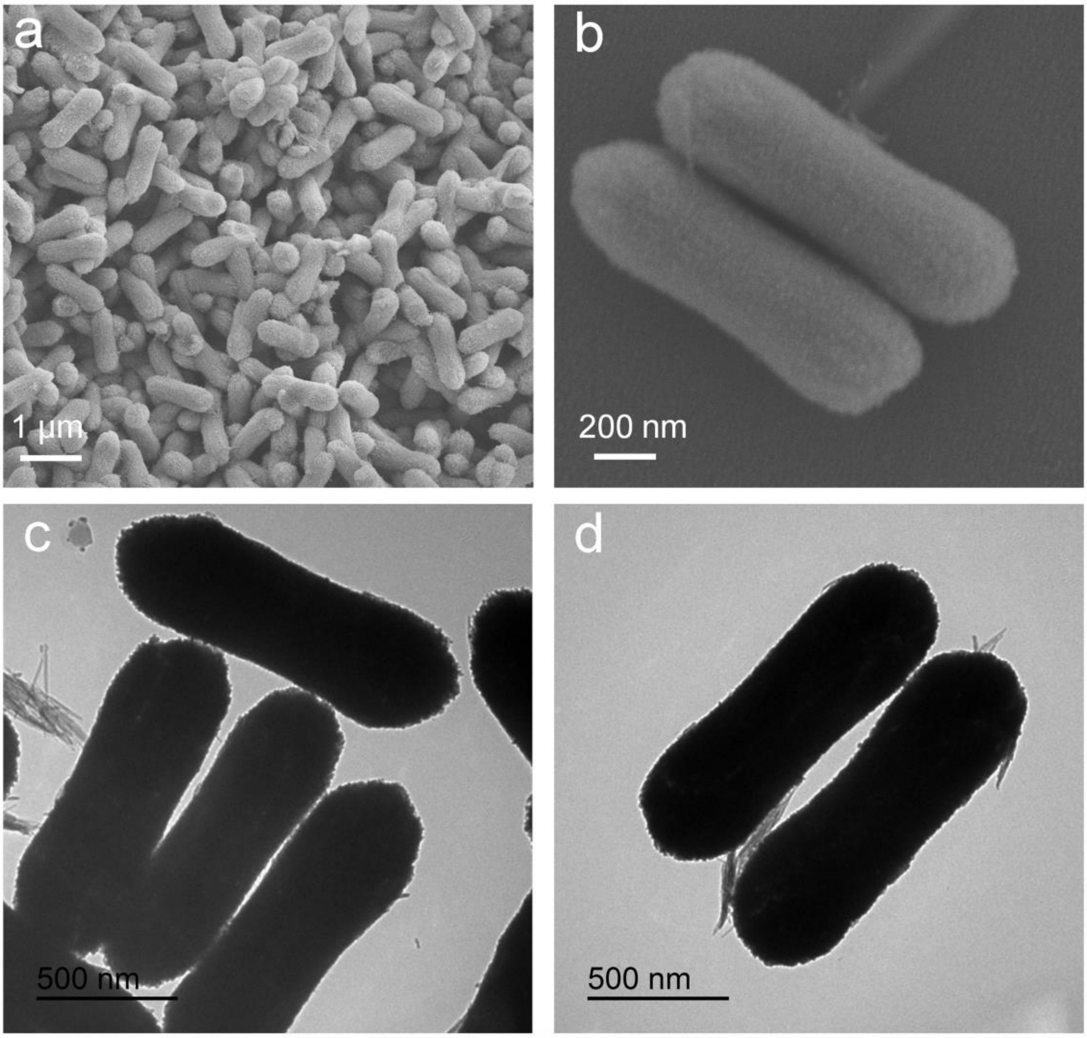


**Figure S5.** (a, b) SEM images of peanut like $Fe_2O_3$. (c, d) TEM images of peanut like $Fe_2O_3$.

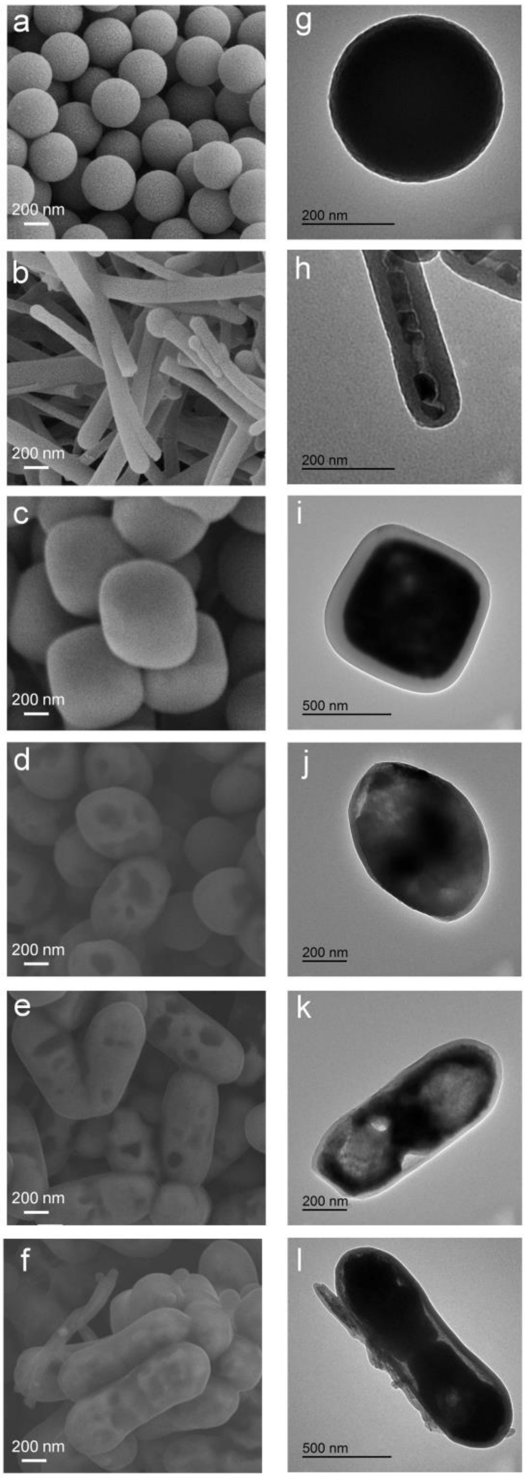


**Figure S6.** SEM images of (a)$SiO_2$@CNSs (b) $MnO_x$@CNRs, (c) $FeO_x$@CNBs, (d) $FeO_x$@CNEs, (e) $FeO_x$@CNCs, and (f) $FeO_x$@CNPs. TEM images of (g)$SiO_2$@CNSs (h) $MnO_x$@CNRs, (i) $FeO_x$@CNBs, (j) $FeO_x$@CNEs, (k) $FeO_x$@CNCs, and (l) $FeO_x$@CNPs.

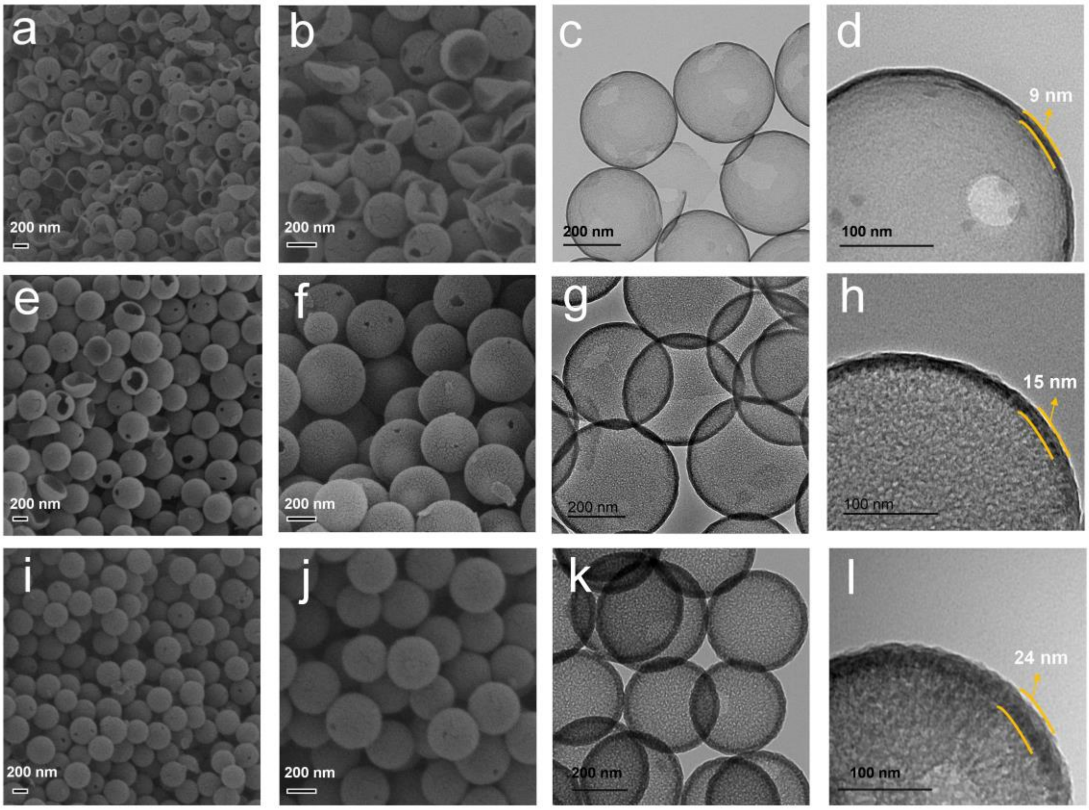


**Figure S7.** SEM images of (a, b) HCNSs-0.2, (e, f) HCNSs-0.4, (i, j) HCNSs-0.6. TEM images of (c, d) HCNSs-0.2, (g, h) HCNSs-0.4, (k, l) HCNSs-0.6.

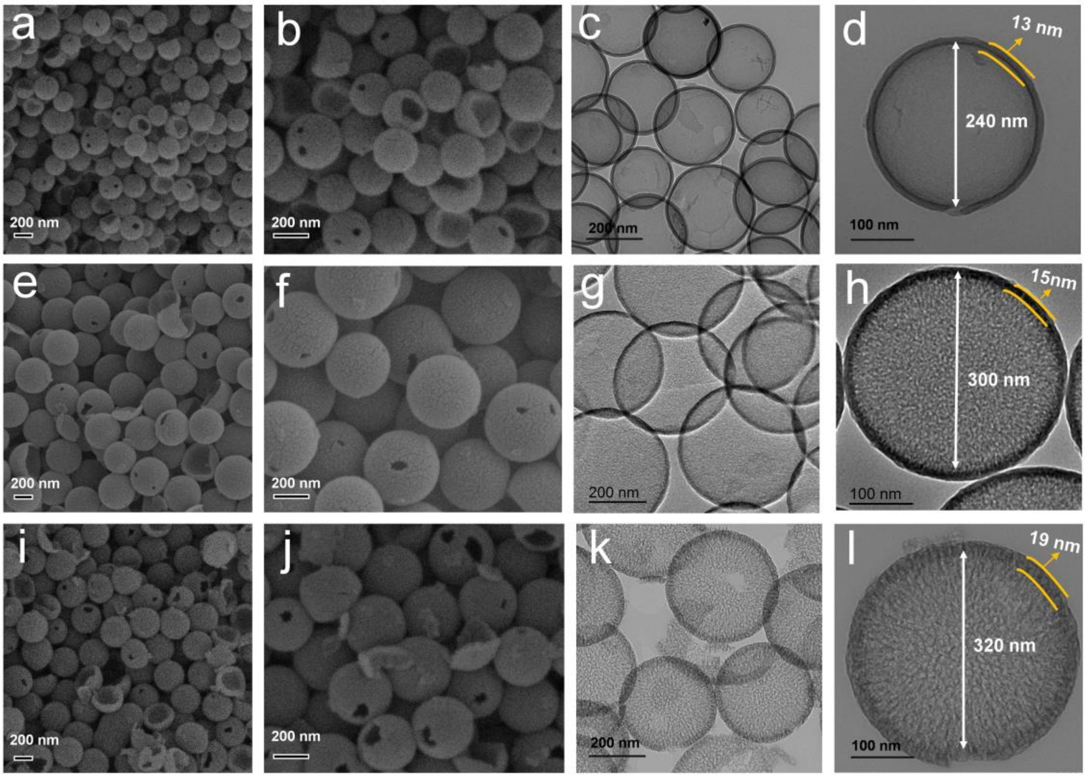


**Figure S8.** SEM images of (a, b) HCNSs-0.1, (e, f) HCNSs-0.4, (i, j) HCNSs-0.8. TEM images of (c, d) HCNSs-0.1, (g, h) HCNSs-0.4, (k, l) HCNSs-0.8.

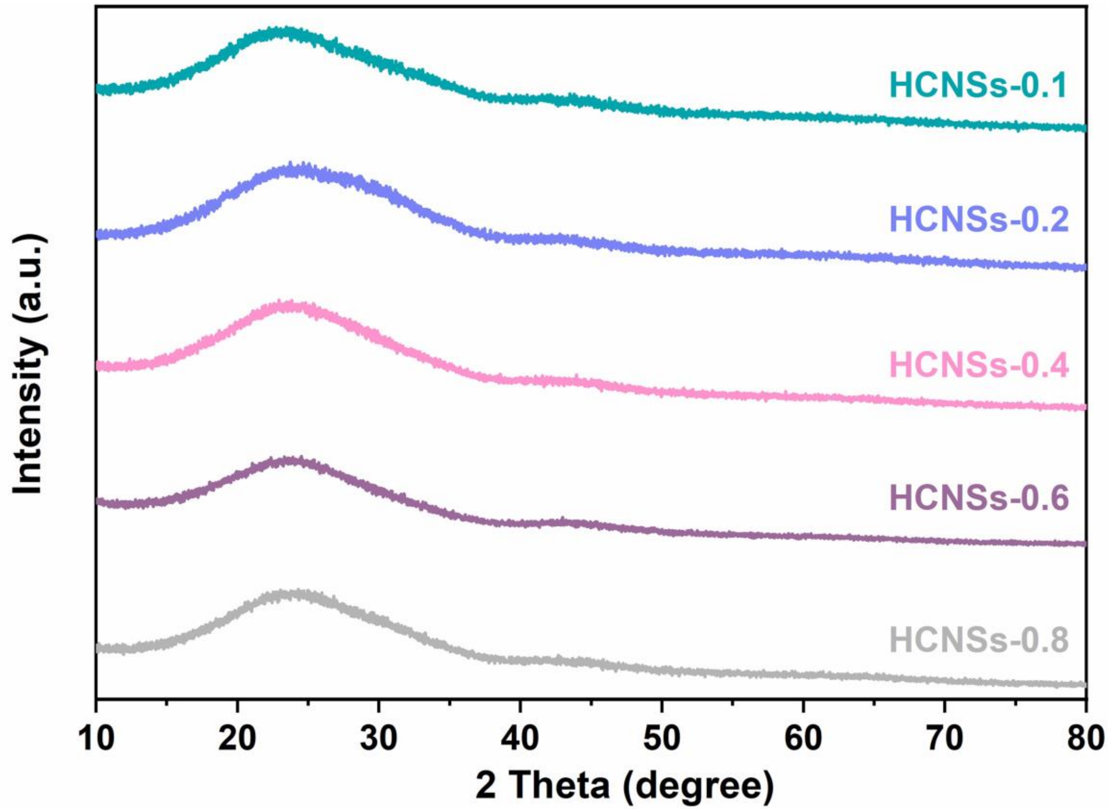


**Figure S9.** XRD patterns of HCNSs-0.1, HCNSs-0.2, HCNSs-0.4, HCNSs-0.6, and HCNSs-0.8.

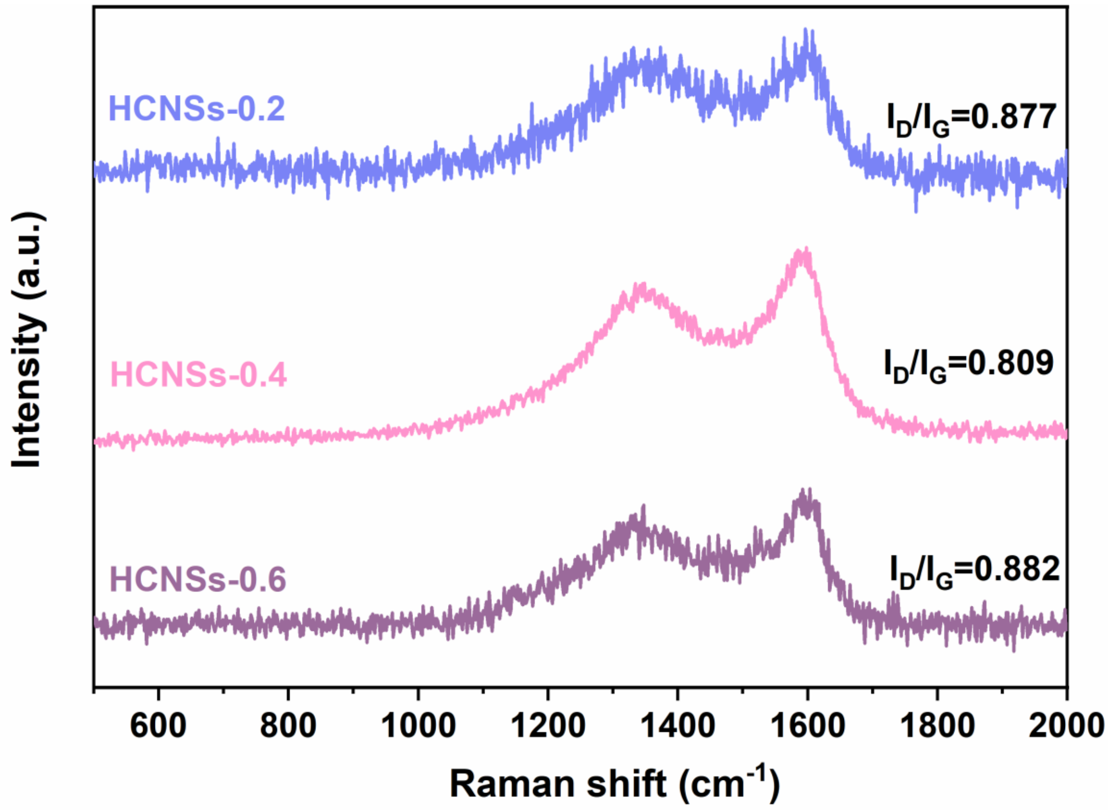


**Figure S10.** Raman plots of HCNSs-0.2, HCNSs-0.4, and HCNSs-0.6.

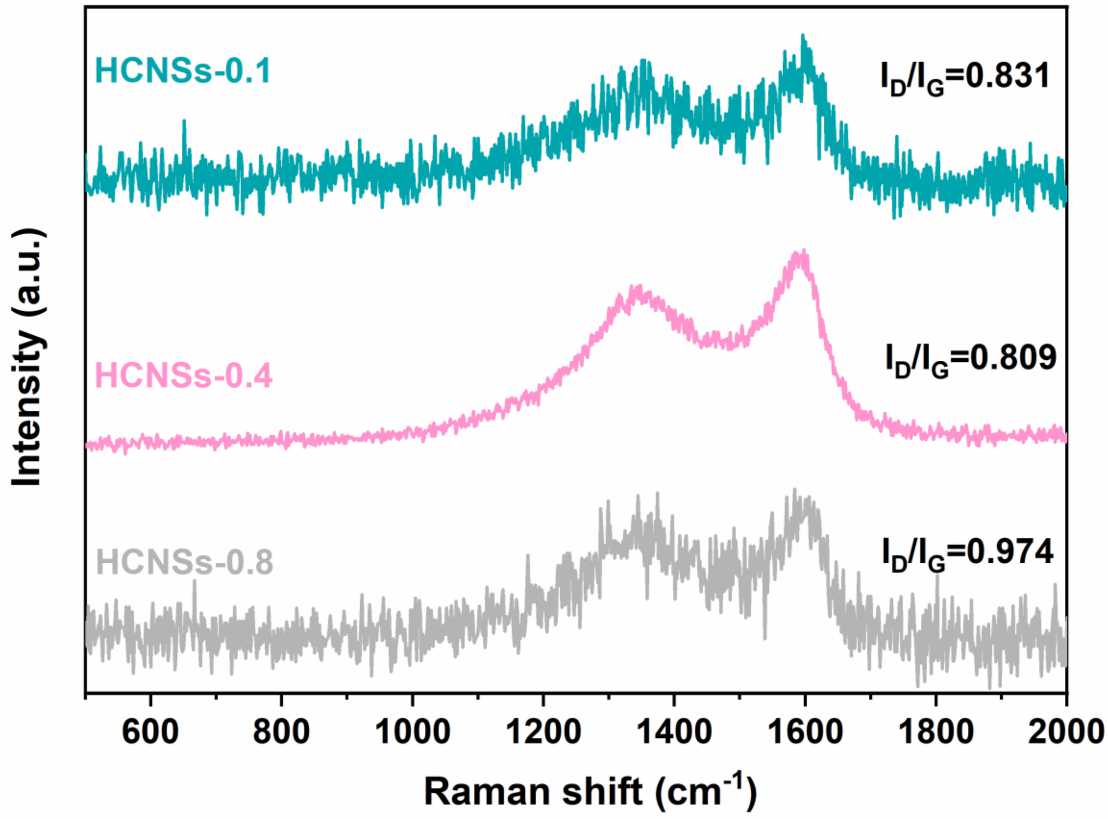


**Figure S11.** Raman plots of HCNSs-0.1, HCNSs-0.4, and HCNSs-0.8.

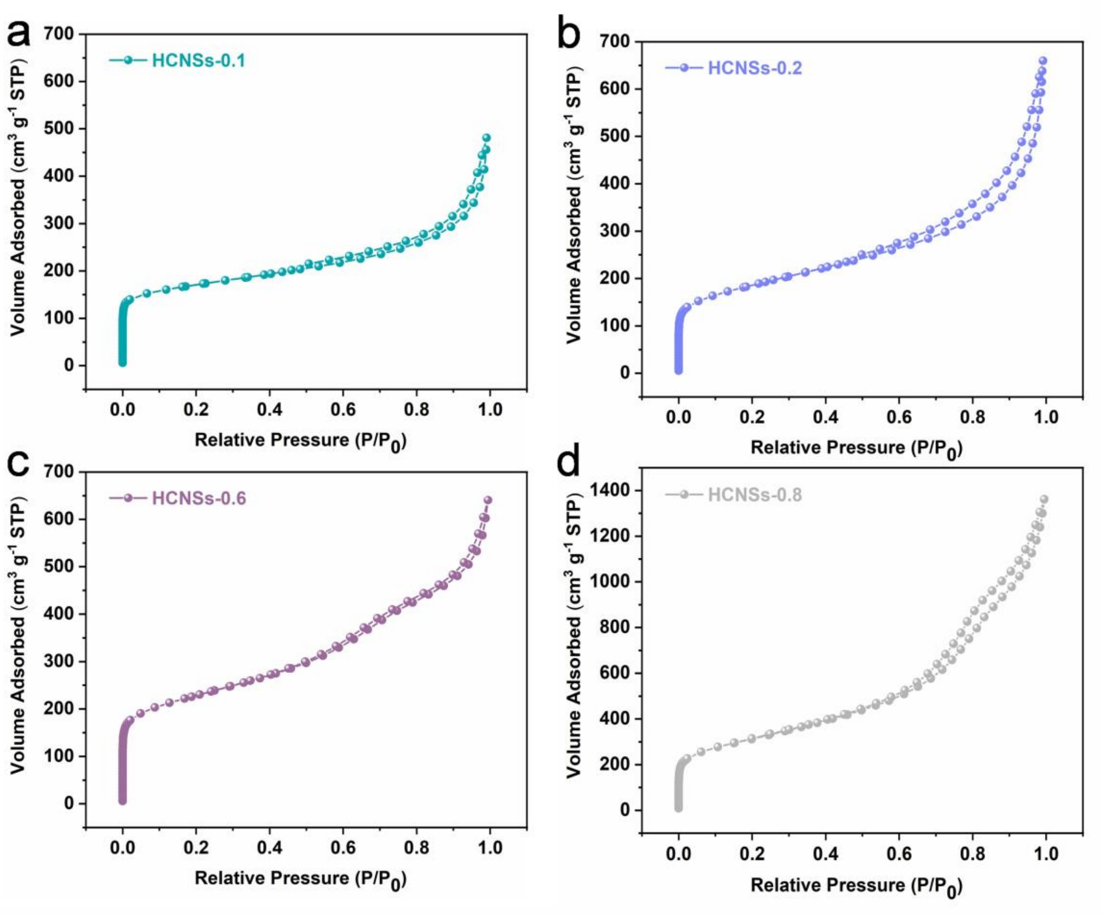


**Figure S12.** $N_2$ adsorption-desorption isotherms of (a) HCNSs-0.1, (b) HCNSs-0.2, (c) HCNSs-0.6, and (d) HCNSs-0.8.

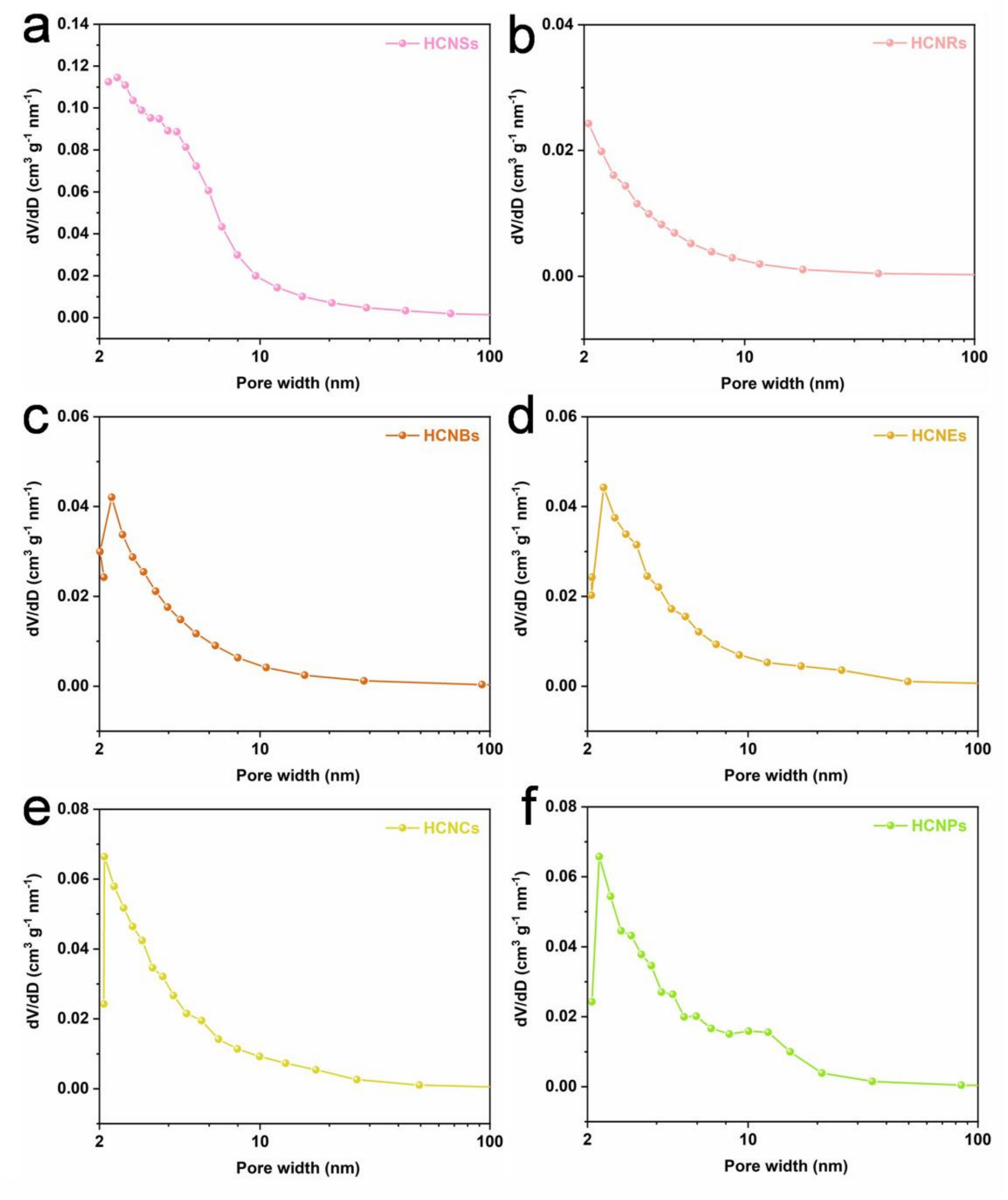


**Figure S13.** BJH pore size distribution of (a) HCNSs, (b) HCNRs, (c) HCNBs, (d) HCNEs, (e) HCNCs, (f) HCNPs.

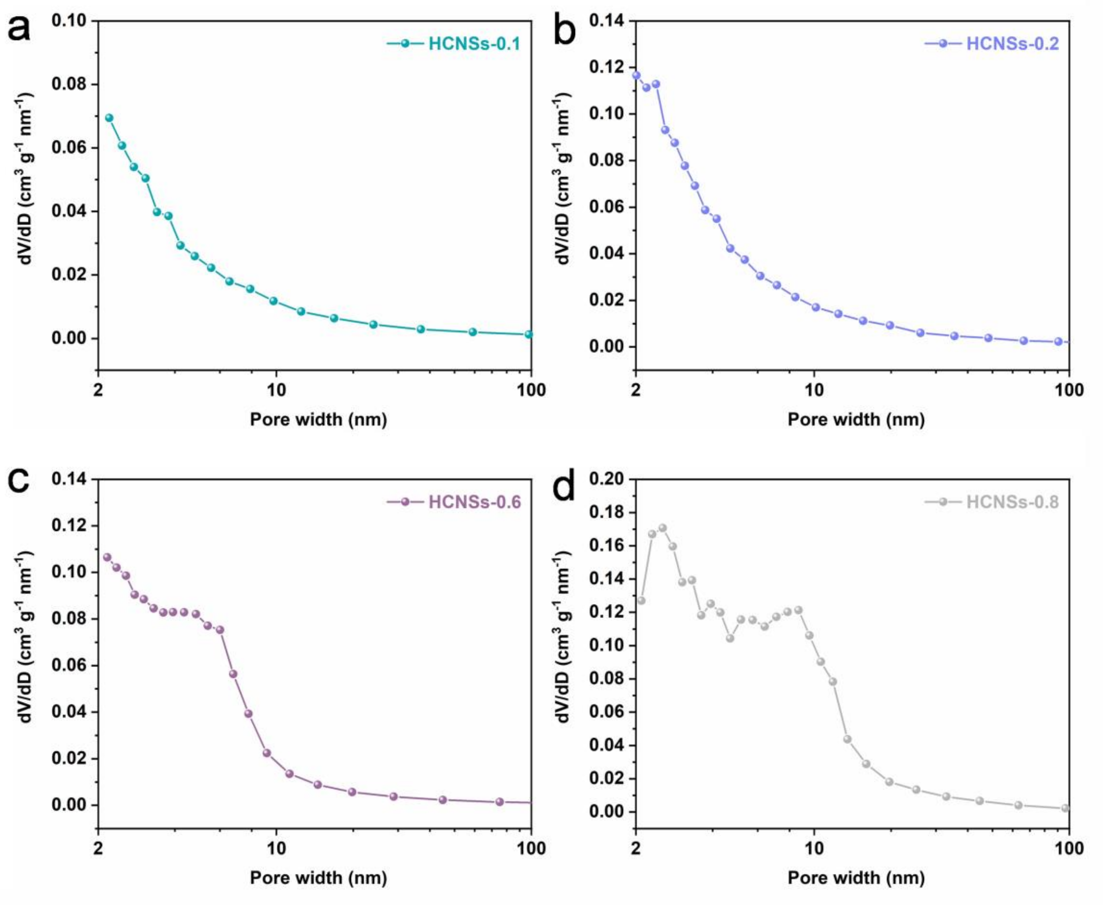


**Figure S14.** BJH pore size distribution of (a) HCNSs-0.1, (b) HCNSs-0.2, (c) HCNSs-0.6, and (d) HCNSs-0.8.

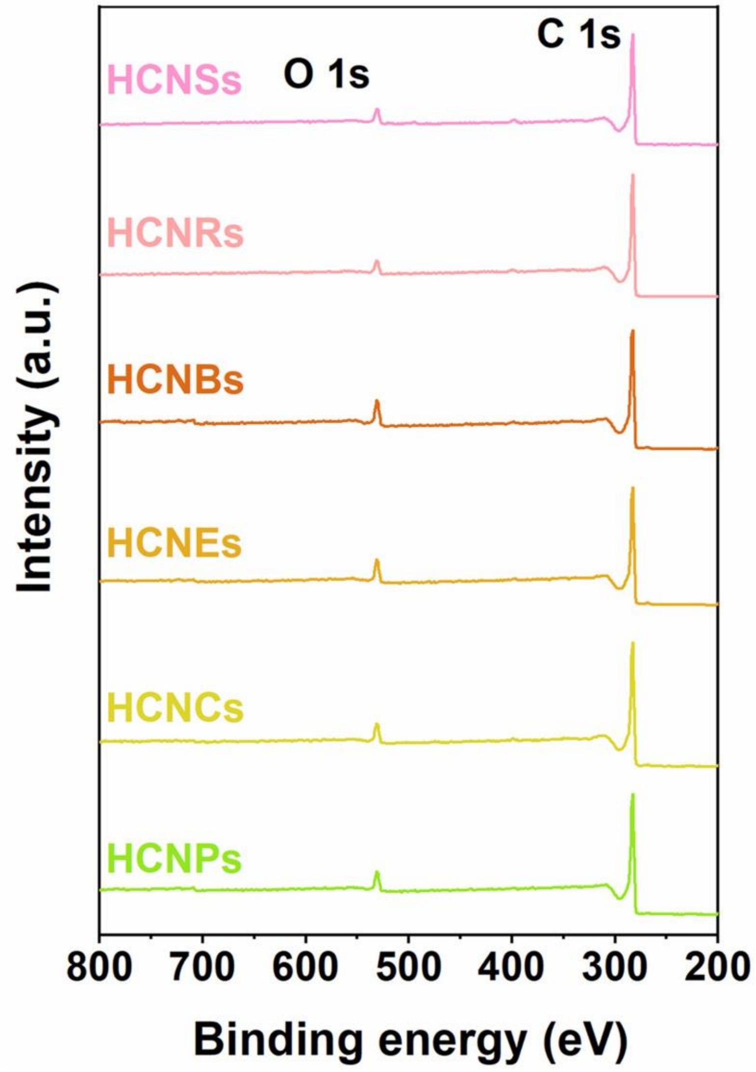


**Figure S15.** XPS spectra of HCNSs, HCNRs, HCNBs, HCNEs, HCNCs, and HCNPs full spectrum.

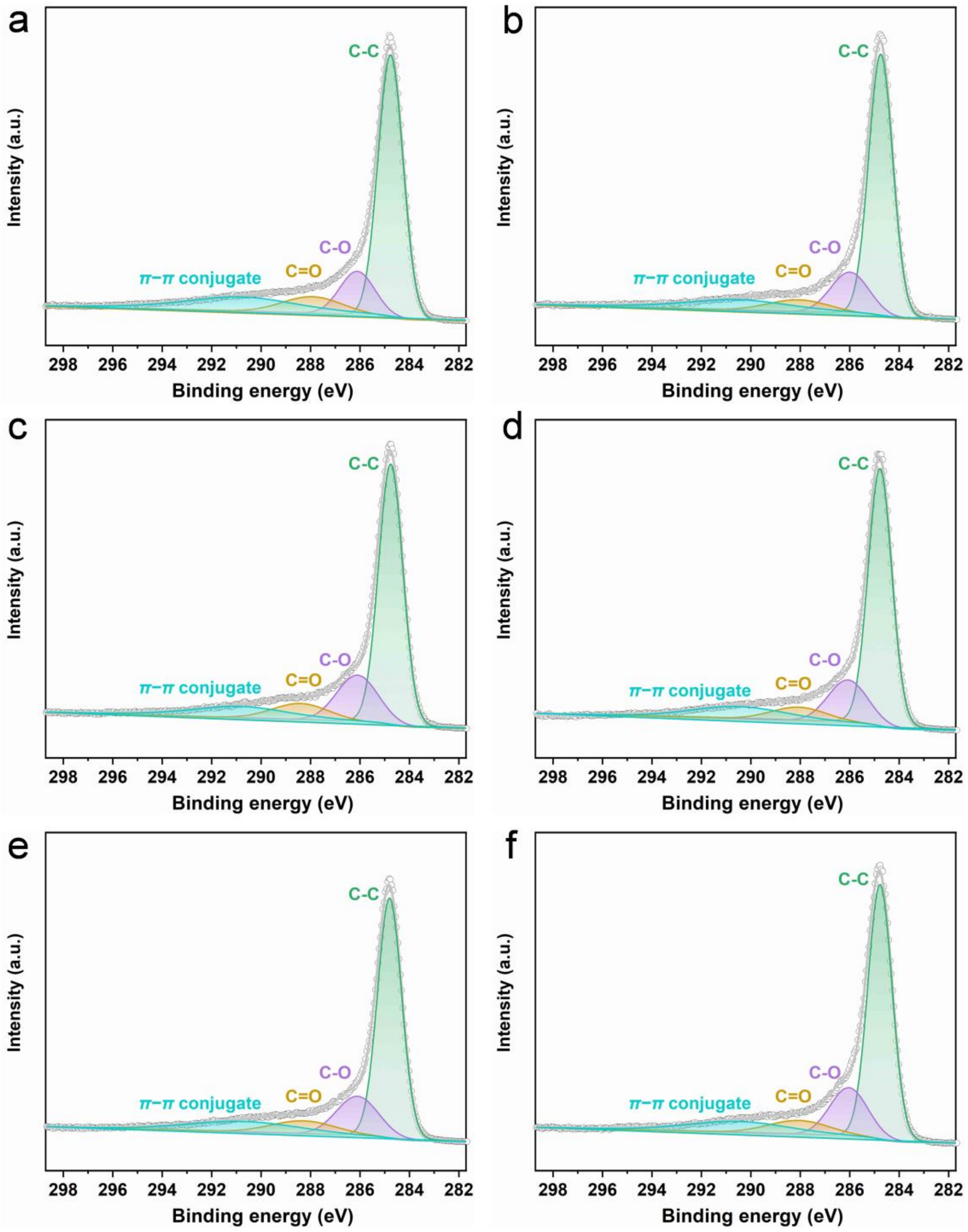


**Figure S16.** High-resolution spectra of C 1s for (a) HCNSs, (b) HCNRs, (c) HCNBs, (d) HCNEs, (e) HCNCs, and (f) HCNPs.

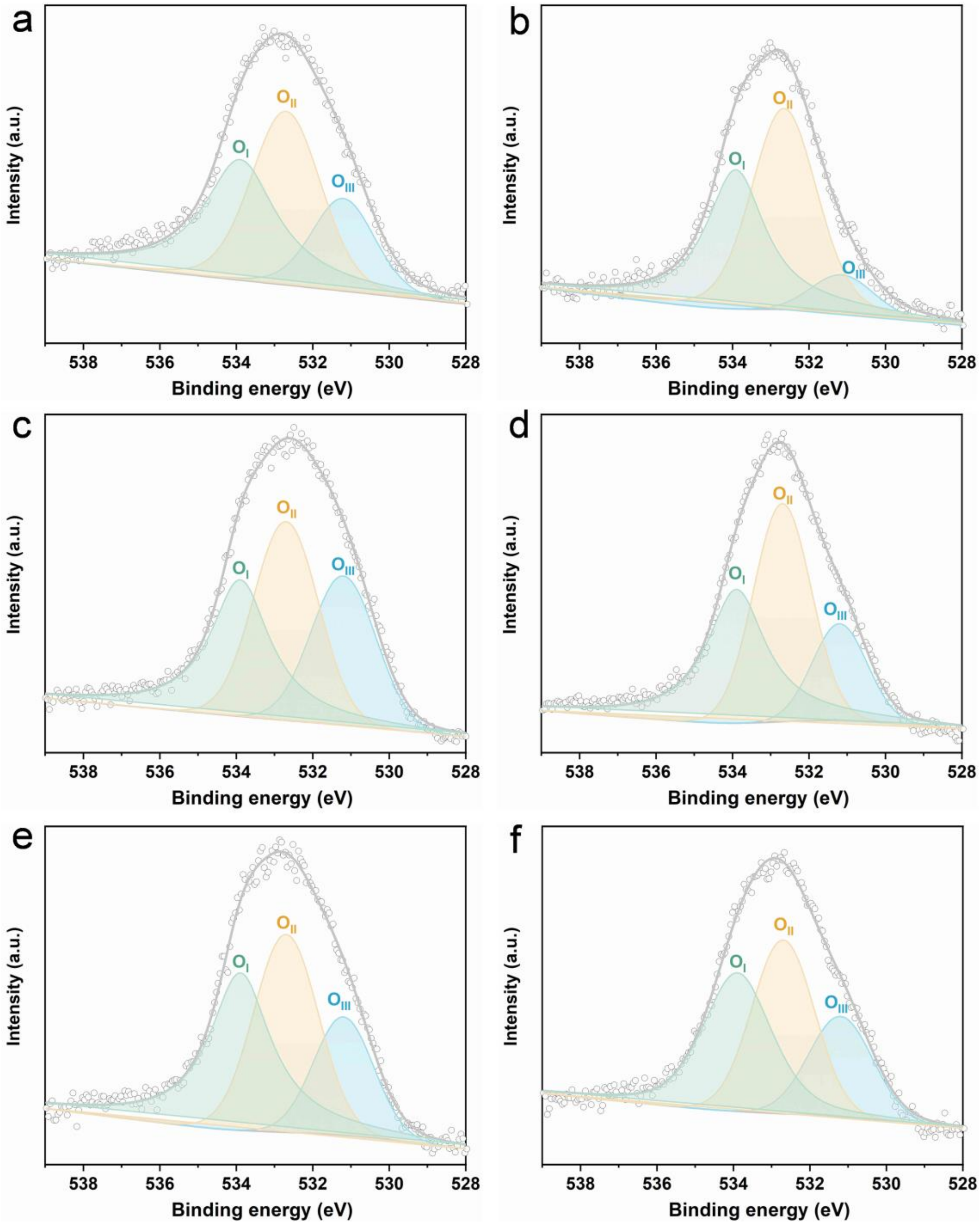


**Figure S17.** High-resolution spectra of O 1s for (a) HCNSs, (b) HCNRs, (c) HCNBs, (d) HCNEs, (e) HCNCs, and (f) HCNPs.

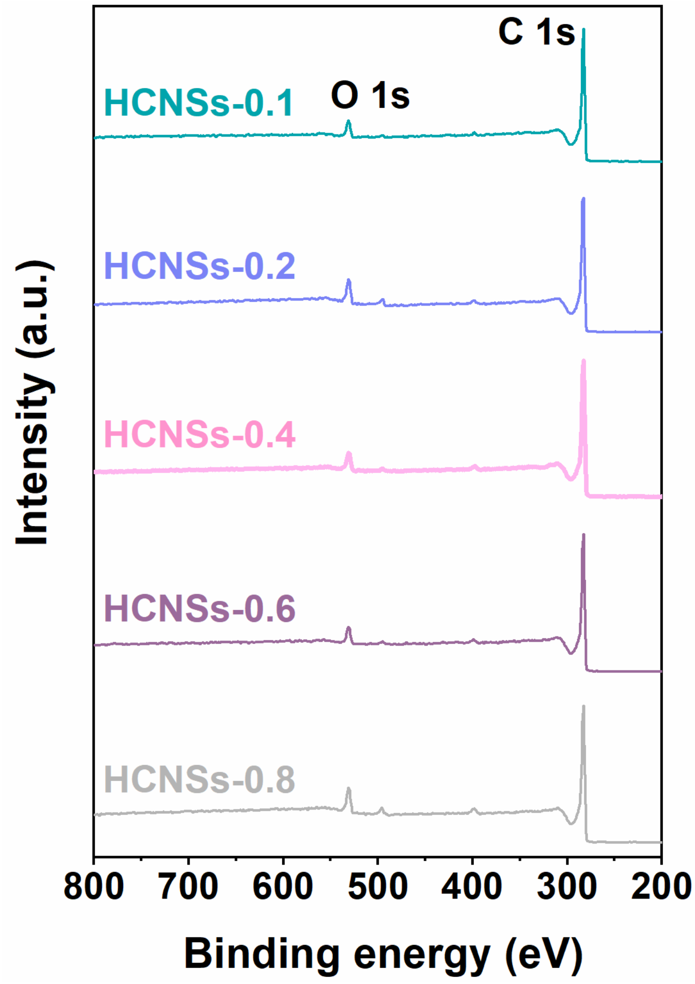


**Figure S18.** XPS spectra of HCNSs-0.1, HCNSs-0.2, HCNSs-0.4, HCNSs-0.6, and HCNSs-0.8 full spectrum.

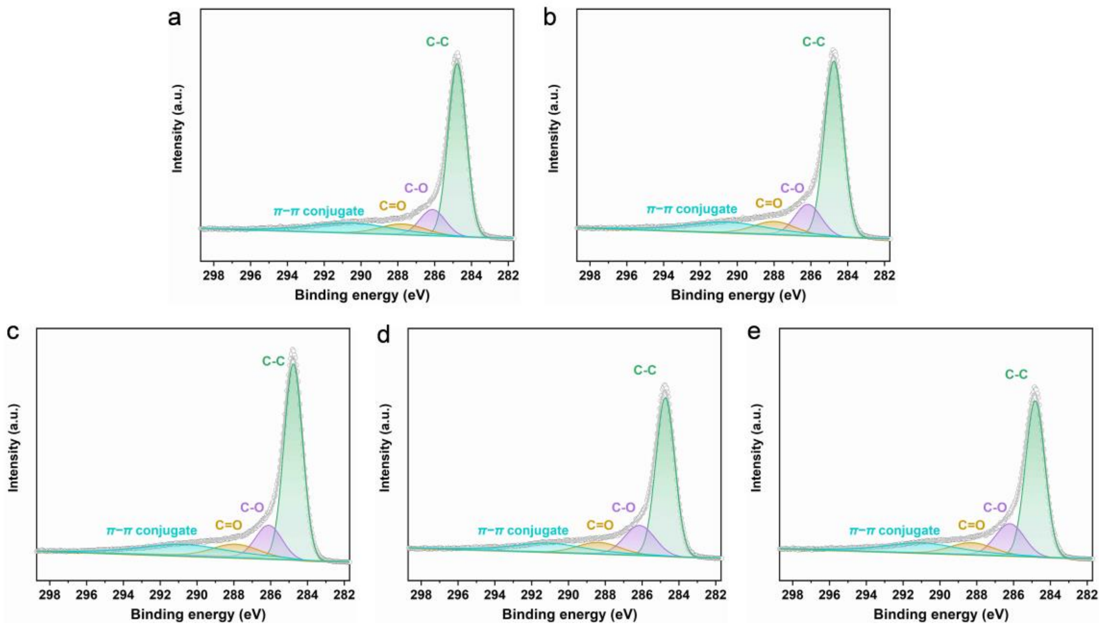


**Figure S19.** High-resolution spectra of C 1s for (a) HCNSs-0.1, (b) HCNSs-0.2, (c) HCNSs-0.4, (d) HCNSs-0.6, and (e) HCNSs-0.8.

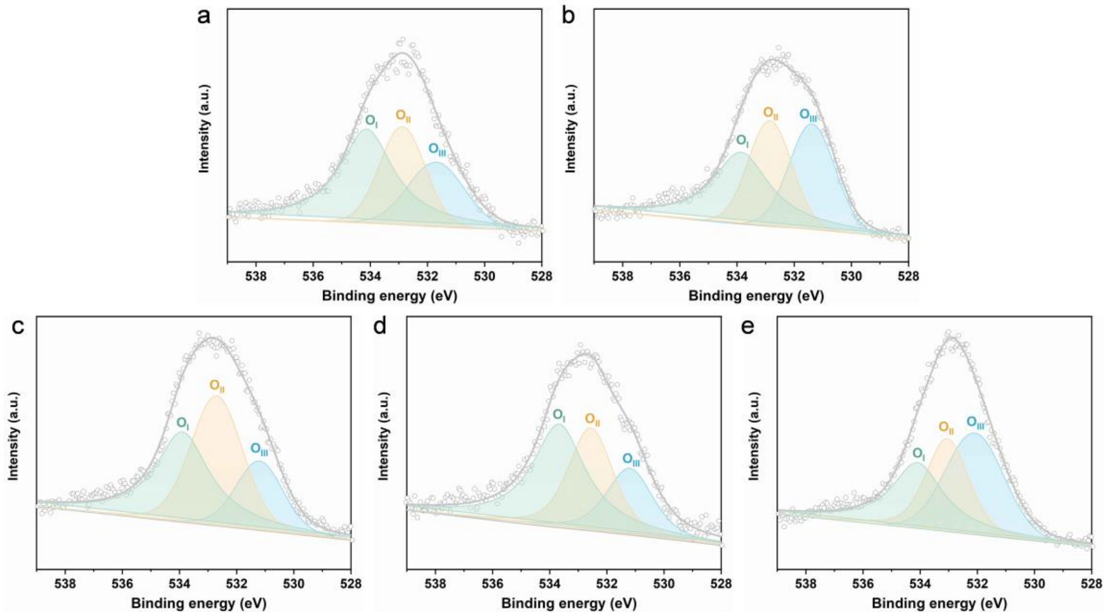


**Figure S20.** High-resolution spectra of O 1s for (a) HCNSs-0.1, (b) HCNSs-0.2, (c) HCNSs-0.4, (d) HCNSs-0.6, and (e) HCNSs-0.8.

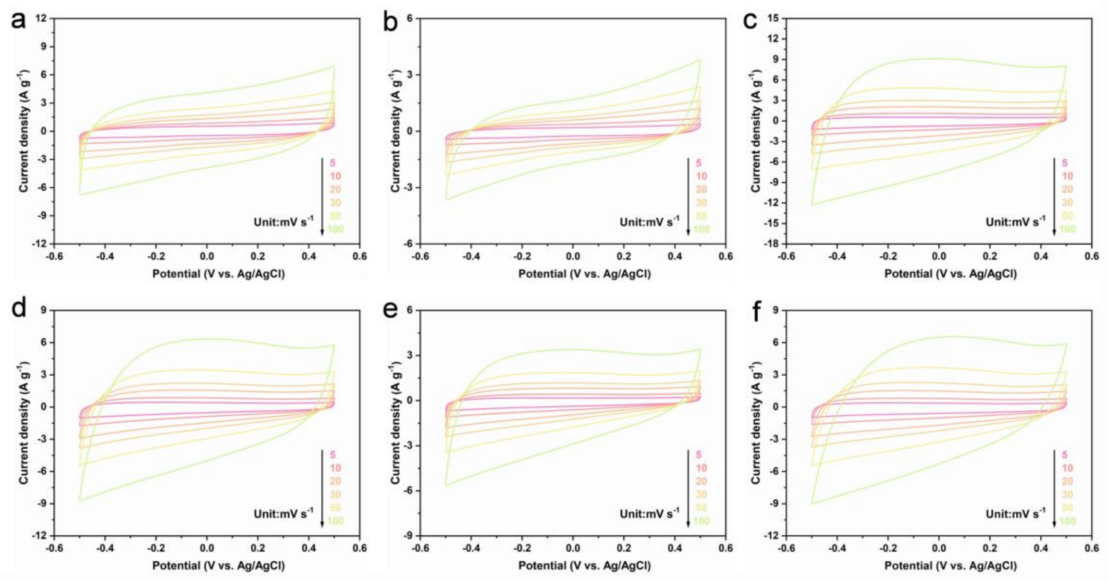


**Figure S21.** CV curves of (a) HCNSs, (b) HCNRs, (c) HCNBs, (d) HCNEs, (e) HCNCs, and (f) HCNPs at different scan rates.

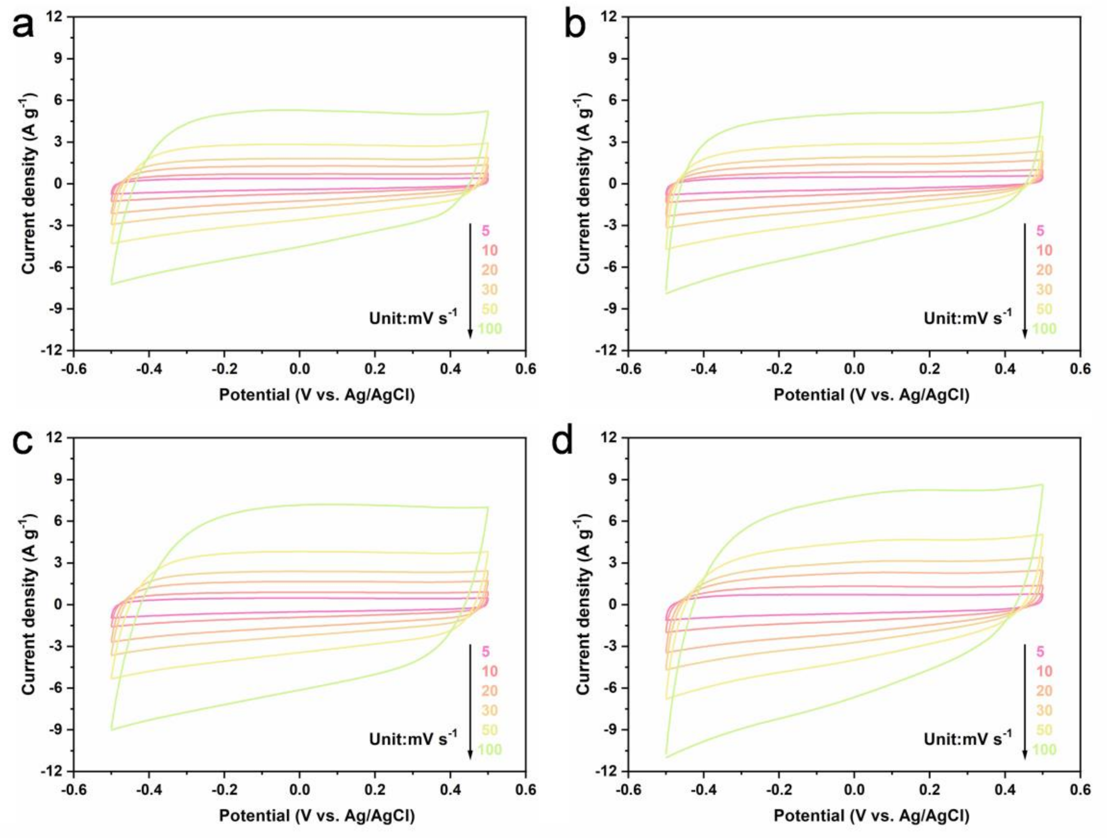


**Figure S22.** CV curves of (a) HCNSs-0.1, (b) HCNSs-0.2, (c) HCNSs-0.6, and (d) HCNSs-0.8 at different scan rates.

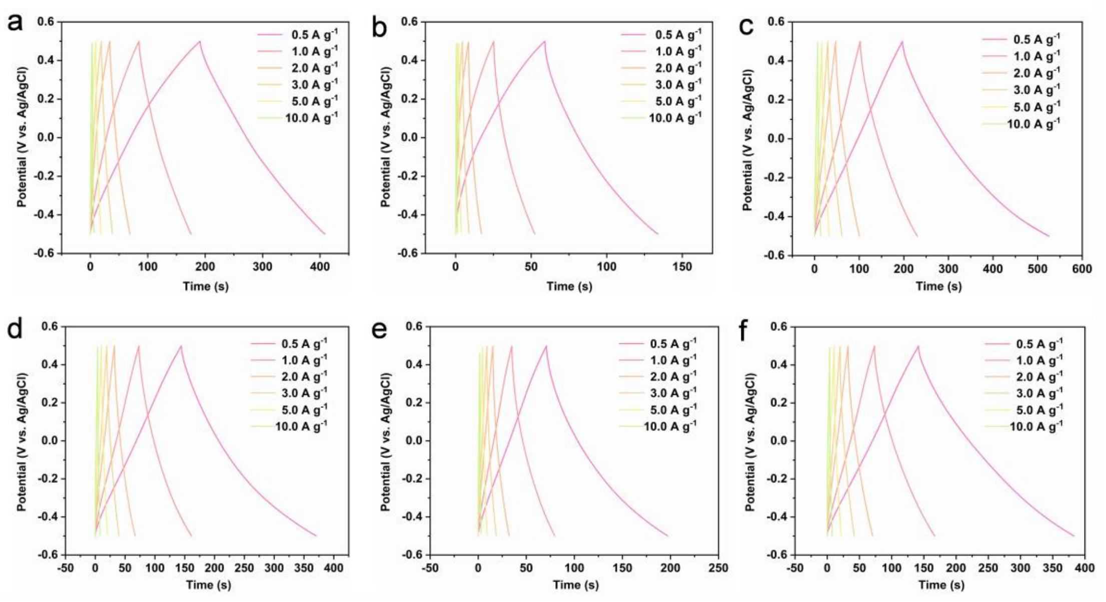


**Figure S23.** GCD curves of (a) HCNSs, (b) HCNRs, (c) HCNBs, (d) HCNEs, (e) HCNCs, and (f) HCNPs at different current densities.

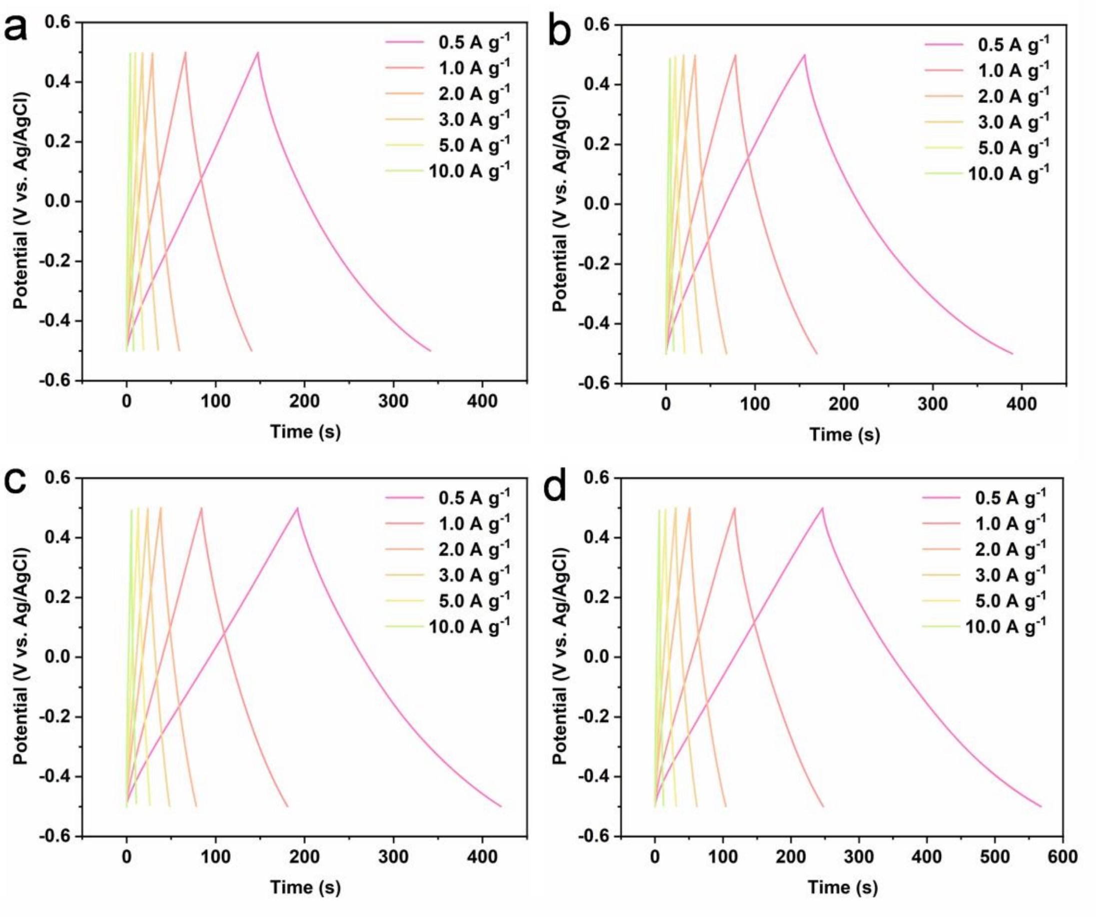


**Figure S24.** GCD curves of (a) HCNSs-0.1, (b) HCNSs-0.2, (c) HCNSs-0.6, and (d) HCNSs-0.8 at different current densities.

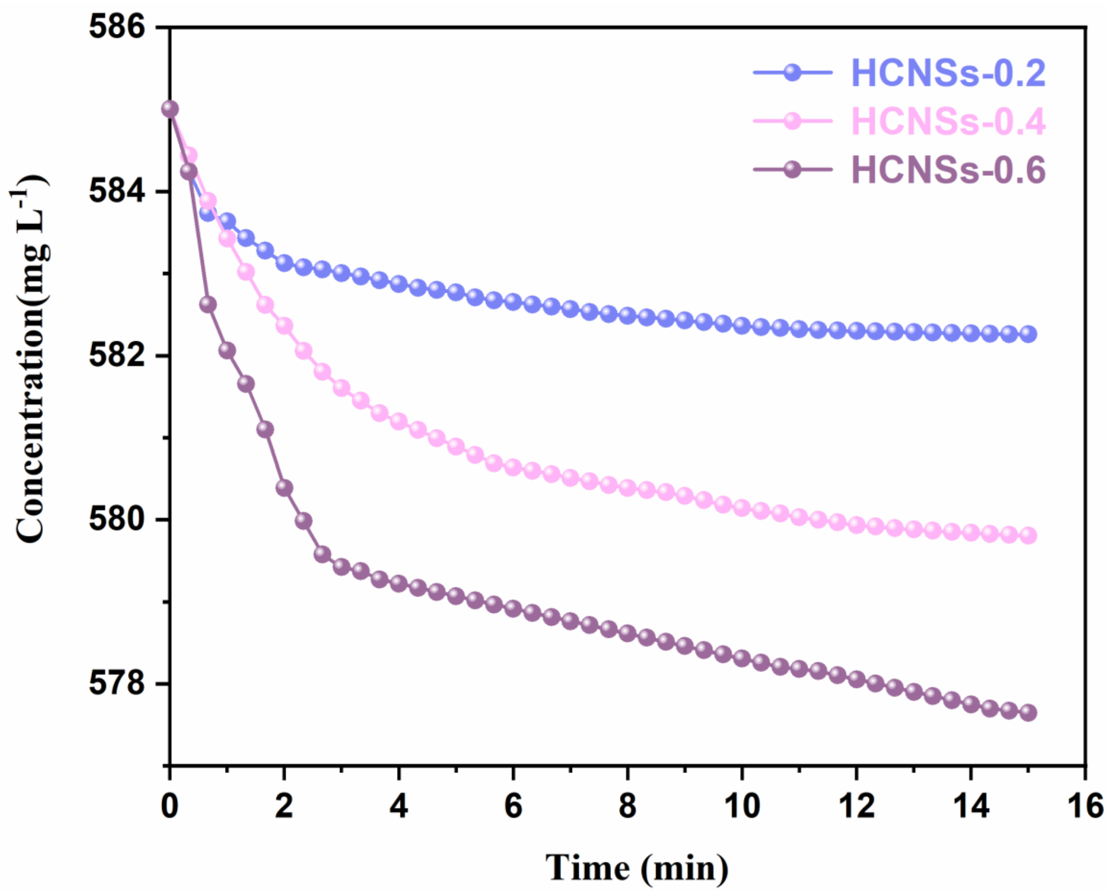


**Figure S25.** NaCl concentration variations of HCNSs-0.2, HCNSs-0.4, and HCNSs-0.6 in 10.0 mM NaCl.

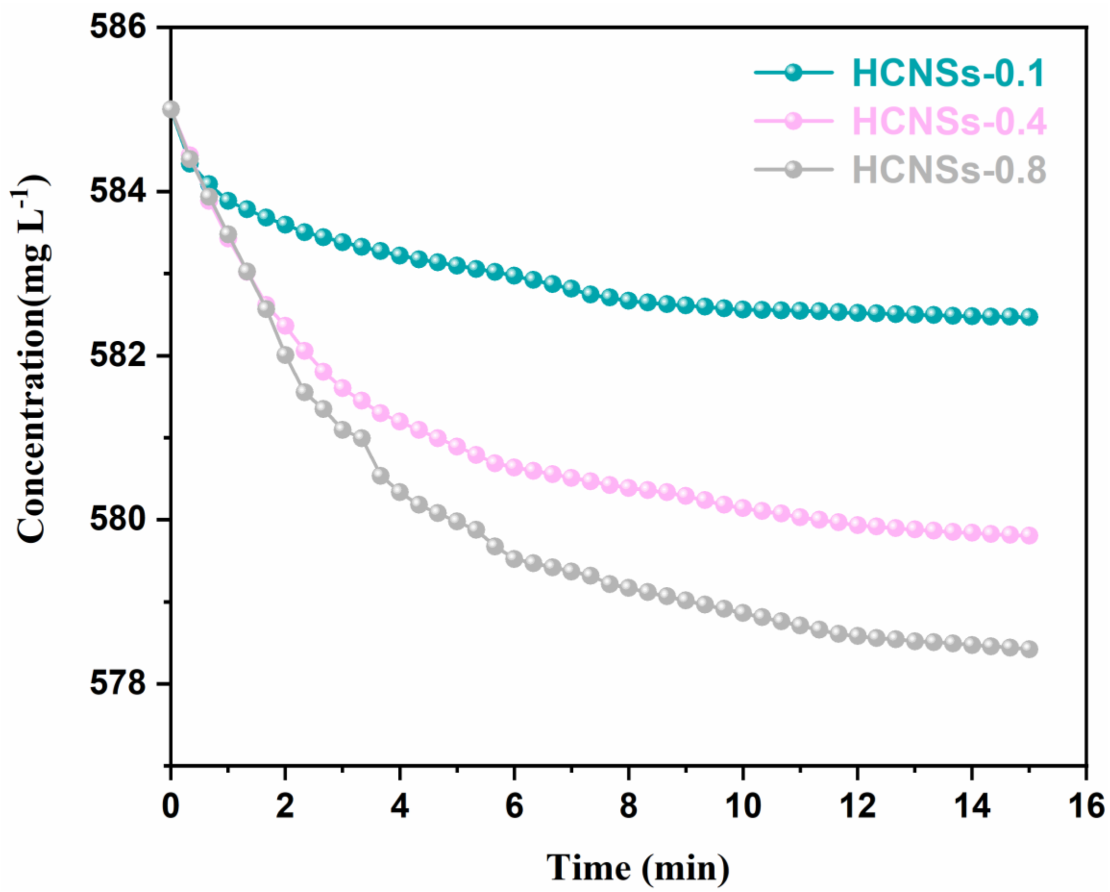


**Figure S26.** NaCl concentration variations of HCNSs-0.1, HCNSs-0.4, and HCNSs-0.8 in 10.0 mM NaCl.

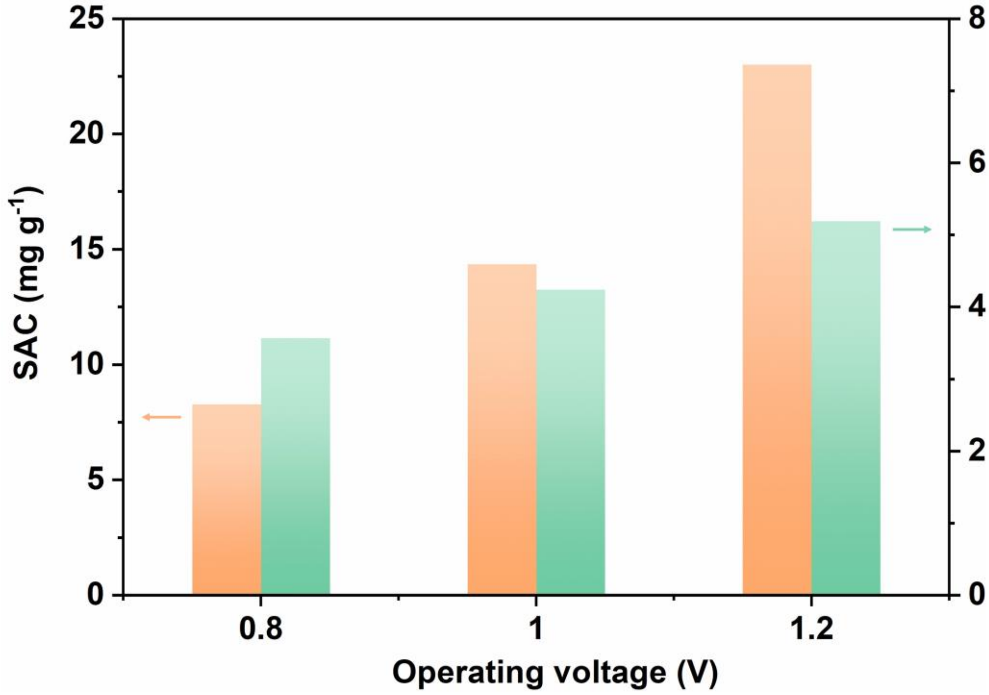


**Figure S27.** Desalination capacity and maximum desalination rate of HCNSs-0.8 at different operating voltage.

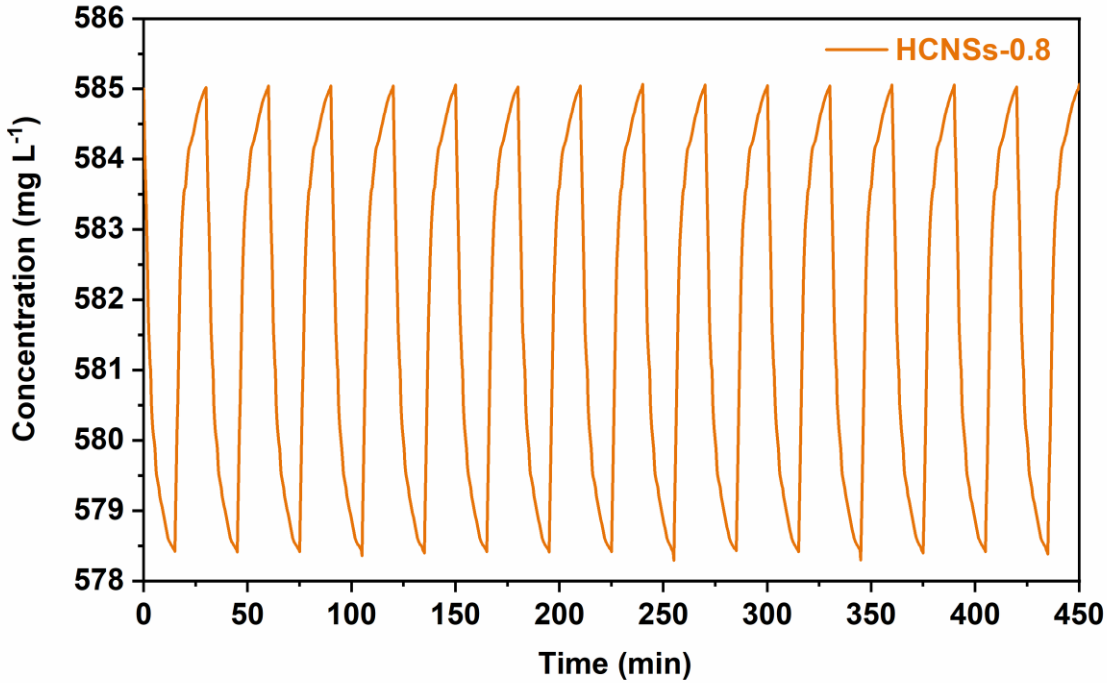


**Figure S28.** Desalination and regeneration experiment for the HCNSs-0.8 electrode.

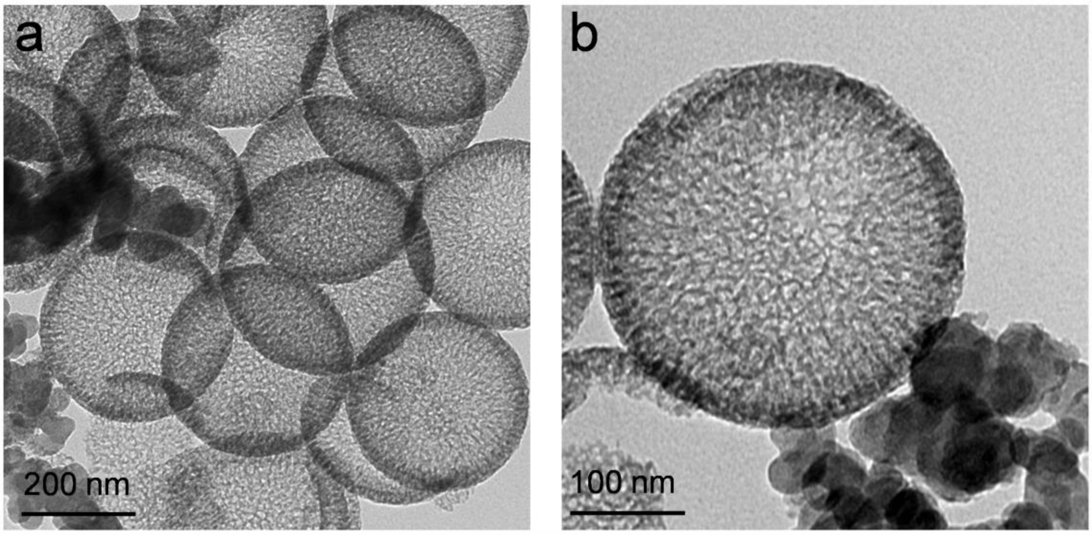


**Figure S29.** TEM images of HMCSs-0.8 after 15 CDI cycles.

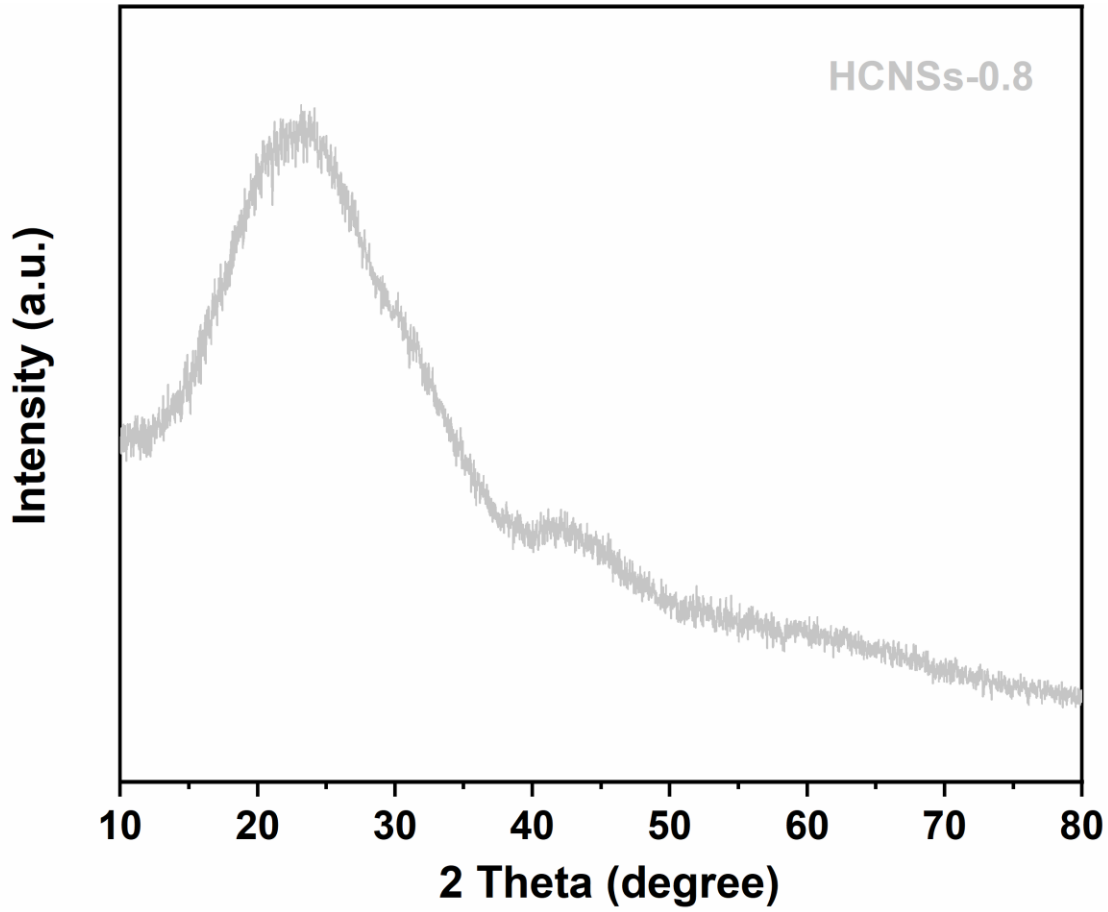


**Figure S30.** XRD pattern of HMCSs-0.8 after 15 CDI cycles.

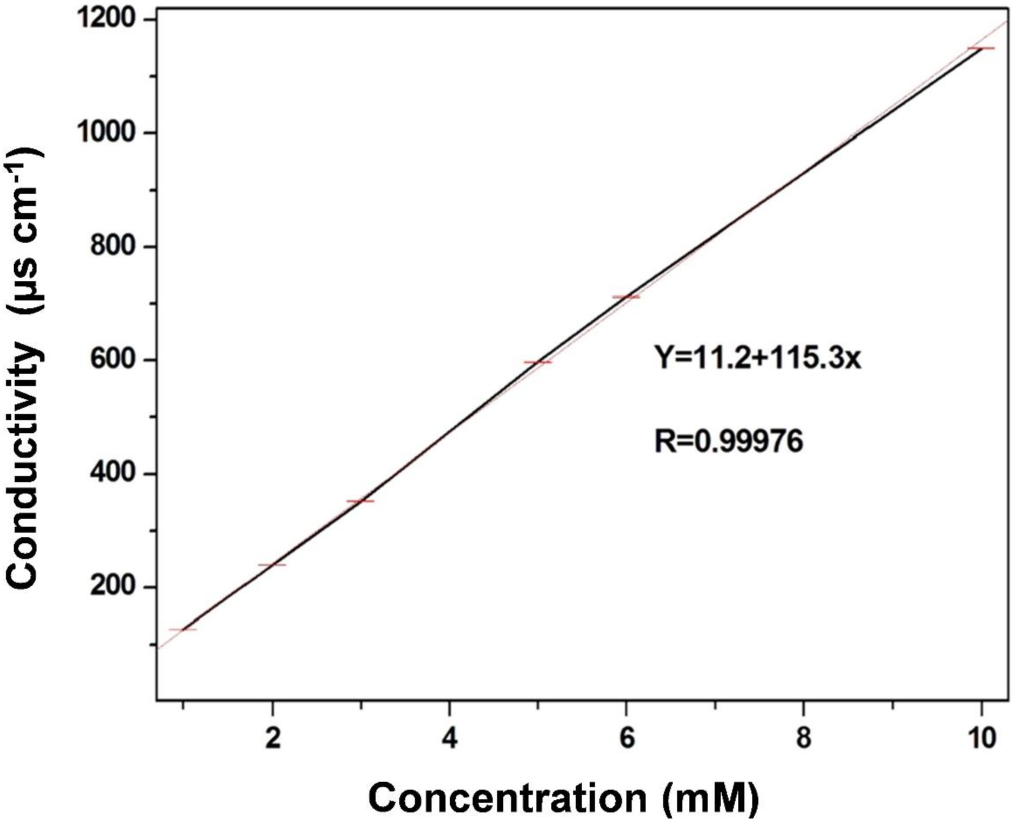


**Figure S31.** The relationship curve between concentration of NaCl solution and conductivity.

**Table S1.** Comparison of material adsorption capacity reported in the literature.

| Electrode material | Operating voltage (V) | Initial NaCl concentration (mg $L^{-1}$) | SAC (mg $g^{-1}$) | Ref. |
|---|---|---|---|---|
| N-HMCSs | 1.6 | 500 | 16.6 | [1] |
| NCPC-900 | 1.6 | 500 | 17.2 | [2] |
| CHS 1 | 1.6 | 250 | 18.8 | [3] |
| HCB | 1.4 | 450 | 21.8 | [4] |
| NPHCS | 1.4 | 500 | 12.95 | [5] |
| HAC-1 | 1.2 | 500 | 29.5 | [6] |
| $TiS_2$@HCSs | 1.2 | 100 | 18.0 | [7] |
| N-CHS | 1.2 | 250 | 13,38 | [8] |
| NSHPC | 1.2 | 500 | 18.71 | [9] |
| A-HCMs | 1.0 | 400 | 14.64 | [10] |
| NC-800 | 1.2 | 58 | 8.52 | [11] |
| $Ni_3Si_2O_5(OH)_4$/C | 1.2 | 584 | 28.7 | [12] |
| Ag/ZCs | 1.2 | 500 | 29.18 | [13] |
| HCNBs | 1.2 | 584 | 14.37 | This work |
| HCNEs | 1.2 | 584 | 10.59 | This work |
| HCNCs | 1.2 | 584 | 9.33 | This work |
| HCNPs | 1.2 | 584 | 10.52 | This work |
| HCNRs | 1.2 | 584 | 9.12 | This work |
| HCNSs-0.1 | 1.2 | 584 | 9.31 | This work |
| HCNSs-0.2 | 1.2 | 584 | 11.53 | This work |
| HCNSs-0.4 | 1.2 | 584 | 18.04 | This work |
| HCNSs-0.6 | 1.2 | 584 | 21.89 | This work |
| HCNSs-0.8 | 1.2 | 584 | 23.01 | This work |

**SAC: salt adsorption capacity**

**Table S2.** Specific surface areas, pore volumes and mean pore diameters of HCNSs, HCNRs, HCNBs, HCNEs, HCNCs, and HCNPs.

| Sample | BET Surface Area ($m^2$ $g^{-1}$) | Pore Volume ($cm^3$ $g^{-1}$) | Pore Size (nm) |
|---|---|---|---|
| HCNSs | 783.84 | 1.03 | 5.26 |
| HCNRs | 342.21 | 0.25 | 2.91 |
| HCNBs | 219.42 | 0.25 | 4.59 |
| HCNEs | 325.78 | 0.38 | 4.60 |
| HCNCs | 412.60 | 0.45 | 4.38 |
| HCNPs | 476.94 | 0.52 | 4.34 |

**Table S3.** Specific surface areas, pore volumes and mean pore diameters of HCNSs-0.1, HCNSs-0.2, HCNSs-0.4, HCNSs-0.6, and HCNSs-0.8.

| Sample | BET Surface Area ($m^2$ $g^{-1}$) | Pore Volume ($cm^3$ $g^{-1}$) | Pore Size (nm) |
|---|---|---|---|
| HCNSs-0.1 | 556.25 | 0.73 | 5.25 |
| HCNSs-0.2 | 628.46 | 0.99 | 6.33 |
| HCNSs-0.4 | 783.84 | 1.03 | 5.26 |
| HCNSs-0.6 | 754.56 | 0.95 | 5.06 |
| HCNSs-0.8 | 1083.20 | 2.01 | 7.44 |

**References**


[1] Y. Li, J. Qi, J. Li, J. Shen, Y. Liu, X. Sun, J. Shen, W. Han, L. Wang, *ACS Sustainable Chemistry & Engineering* **2017**, *5* (8), 6635, https://doi.org/10.1021/acssuschemeng.7b00884.

[2] Y. Li, Y. Liu, J. Shen, J. Qi, J. Li, X. Sun, J. Shen, W. Han, L. Wang, *Desalination* **2018**, *430*, 45, https://doi.org/10.1016/j.desal.2017.12.040.

[3] Z. Y. Leong, H. Y. Yang, *RSC Advances* **2016**, *6* (58), 53542, https://doi.org/10.1039/c6ra06489b.

[4] X. Zang, Z. Fu, D. Wang, Z. Yuan, N. Shi, Z. Yang, Y.-M. Yan, *Journal of Materials Chemistry A* **2022**, *10* (18), 9988, https://doi.org/10.1039/d2ta00611a.

[5] S. Zhao, T. Yan, H. Wang, G. Chen, L. Huang, J. Zhang, L. Shi, D. Zhang, *Applied Surface Science* **2016**, *369*, 460, https://doi.org/10.1016/j.apsusc.2016.02.085.

[6] M. Kim, X. Xu, R. Xin, J. Earnshaw, A. Ashok, J. Kim, T. Park, A. K. Nanjundan, W. A. El-Said, J. W. Yi, J. Na, Y. Yamauchi, *ACS Appl Mater Interfaces* **2021**, *13*(44), 52034, https://doi.org/10.1021/acsami.1c09107.

[7] M. Ezzati, F. Hekmat, S. Shahrokhian, H. E. Unalan, *Desalination* **2022**, *533*, 115766, https://doi.org/10.1016/j.desal.2022.115766.

[8] M. Ding, F.-H. Du, B. Liu, Z. Y. Leong, L. Guo, F. Chen, A. Baji, H. Y. Yang, *FlatChem* **2018**, *7*, 10, https://doi.org/10.1016/j.flatc.2018.01.002.

[9] Y. Huang, J. Yang, L. Hu, D. Xia, Q. Zhang, Y. Liao, H. Li, W. Yang, C. He, D. Shu, *Environmental Science: Nano* **2019**, *6* (5), 1430, https://doi.org/10.1039/c9en00028c.

[10] P. Wang, W. Ma, S. Xue, L. Wang, Y. Chen, Y. Wang, *Separation and Purification Technology* **2021**, *276*, 119336, https://doi.org/10.1016/j.seppur.2021.119336.

[11] N. L. Liu, S. Dutta, R. R. Salunkhe, T. Ahamad, S. M. Alshehri, Y. Yamauchi, C. H. Hou, K. C. Wu, *Sci Rep* **2016**, *6*, 28847, https://doi.org/10.1038/srep28847.

[12] Y. Tang, S. Zheng, S. Cao, F. Yang, X. Guo, S. Zhang, H. Xue, H. Pang, *J Colloid Interface Sci* **2022**, *626*, 1062, https://doi.org/10.1016/j.jcis.2022.07.034.

[13] H. Zhang, W. Zhang, J. Shen, Y. Li, X. Yan, J. Qi, X. Sun, J. Shen, W. Han, L. Wang, J. Li, *Desalination* **2020**, *473*, 114173 https://doi.org/10.1016/j.desal.2019.114173.
